# The Opening of Connexin 43 Hemichannels Alters Hippocampal Astrocyte Function and Neuronal Survival in Prenatally LPS-Exposed Adult Offspring

**DOI:** 10.3389/fncel.2019.00460

**Published:** 2019-10-11

**Authors:** Carolina E. Chávez, Juan E. Oyarzún, Beatriz C. Avendaño, Luis A. Mellado, Carla A. Inostroza, Tanhia F. Alvear, Juan A. Orellana

**Affiliations:** Departamento de Neurología, Facultad de Medicina, Escuela de Medicina and Centro Interdisciplinario de Neurociencias, Pontificia Universidad Católica de Chile, Santiago, Chile

**Keywords:** neuroinflammation, hemichannel, connexin, glia, pannexin

## Abstract

Clinical evidence has revealed that children born from mothers exposed to viral and bacterial pathogens during pregnancy are more likely to suffer various neurological disorders including schizophrenia, autism bipolar disorder, major depression, epilepsy, and cerebral palsy. Despite that most research has centered on the impact of prenatal inflammation in neurons and microglia, the potential modifications of astrocytes and neuron-astrocyte communication have received less scrutiny. Here, we evaluated whether prenatally LPS-exposed offspring display alterations in the opening of astrocyte hemichannels and pannexons in the hippocampus, together with changes in neuroinflammation, intracellular Ca^2+^ and nitric oxide (NO) signaling, gliotransmitter release, cell arborization, and neuronal survival. Ethidium uptake recordings revealed that prenatal LPS exposure enhances the opening of astrocyte Cx43 hemichannels and Panx1 channels in the hippocampus of adult offspring mice. This enhanced channel activity occurred by a mechanism involving a microglia-dependent production of IL-1β/TNF-α and the stimulation of p38 MAP kinase/iNOS/[Ca^2+^]_i_-mediated signaling and purinergic/glutamatergic pathways. Noteworthy, the activity of Cx43 hemichannels affected the release of glutamate, [Ca^2+^]_i_ handling, and morphology of astrocytes, whereas also disturbed neuronal function, including the dendritic arbor and spine density, as well as survival. We speculate that excitotoxic levels of glutamate triggered by the activation of Cx43 hemichannels may contribute to hippocampal neurotoxicity and damage in prenatally LPS-exposed offspring. Therefore, the understanding of how astrocyte-neuron crosstalk is an auspicious avenue toward the development of broad treatments for several neurological disorders observed in children born to women who had a severe infection during gestation.

## Introduction

Environmental factors during early development have a crucial impact on brain function, causing individual differences that could lead to behavioral alteration and increased risk for neurological diseases over the lifetime (Faa et al., 2016). One of the early experiences that affect the brain outcome is the maternal infection, which impairs the complex immune harmony between the maternal and fetal environments, leading to a disrupted immune profile in the developing brain (Gumusoglu and Stevens, 2019). Indeed, clinical evidence has revealed that children born from mothers exposed to viral and bacterial pathogens during pregnancy are more likely to suffer various neurological disorders such as schizophrenia, autism bipolar disorder, major depression, epilepsy, and cerebral palsy (Bergdolt and Dunaevsky, 2018). Most of these epidemiological data have been reproduced in rodent models linked to the administration of lipopolysaccharide (LPS) during gestation (Boksa, 2010).

Although the offspring from LPS-exposed pregnant rodents displays a wide spectrum of brain abnormalities, including behavioral and cognitive changes, anatomical abnormalities, altered synaptic transmission, and immune disturbances (Golan et al., 2005; Rousset et al., 2006; Escobar et al., 2011), the involved mechanisms remain unknown. Moreover, most research has centered on the impact of prenatal inflammation in neurons and microglia, however, the potential modifications of astrocytes and neuron-astrocyte communication have received less scrutiny. Astrocytes encompass the most ubiquitous glial cell type and are endowed with the ability to sense neuronal function and react to it by releasing biomolecules termed “gliotransmitters” (e.g., glutamate, ATP, and D-serine) (Perea et al., 2009). Brain function is highly dependent on astrocytes, as they govern the energy supply to neurons (lactate) along with controlling the homeostatic balance of extracellular pH, neurotransmitters and ions, as well as modulating the redox response and intracellular Ca^2+^ concentration ([Ca^2+^]_i_) signaling (Santello et al., 2019). During brain disease, astrocytes undergo a protective cell reaction called “reactive astrogliosis,” however, when damage turns persistent, this response could disturb astrocyte-to-neuron communication and facilitate the recruitment of the innate immune response (Pekny and Pekna, 2014).

Despite that is known that prenatal LPS exposure triggers reactive astrogliosis (Hao et al., 2010; Zager et al., 2015), the signaling that shed light on this phenomenon and whether other astrocytic properties (e.g., gliotransmitter release, [Ca^2+^]_i_ dynamics, NO production) are disturbed remain poorly understood. Current studies suggest that cellular cascades associated to hemichannels and pannexons are pivotal for astroglial function and dysfunction (Abudara et al., 2018). Hemichannels are plasma membrane channels composed by the oligomerization of six connexin monomers around a central pore that permit the diffusion of ions and small molecules, acting as a signaling route for communication between the cytoplasmic and extracellular compartments (Abudara et al., 2018). On the other hand, pannexins channels or pannexons are made of the oligomerization of pannexins, a three-member protein family with similar secondary and tertiary structures than connexins and with the capacity to form plasma membrane channels (Dahl, 2018).

In astrocytes, hemichannels and pannexons permit the release of gliotransmitters that have been found crucial for synaptic transmission and plasticity, as well as behavior and memory (Stehberg et al., 2012; Ardiles et al., 2014; Chever et al., 2014; Walrave et al., 2016; Meunier et al., 2017). However, during pathological conditions, the permanent activity of these channels has been linked to the homeostatic disturbances occurring in the pathogenesis and progression of multiple diseases (Salameh et al., 2013; Orellana et al., 2016; Leybaert et al., 2017). In a previous study, using neonatal primary cell cultures, we demonstrated that astrocytes obtained from the offspring of LPS-exposed dams show an elevated opening of hemichannels and pannexons (Avendano et al., 2015). Nevertheless, whether prenatal LPS exposure affects the opening of these channels in the adult offspring and the possible impact of this on astrocyte function and neuronal survival is still ignored. Here, by performing studies in the stratum radiatum of the hippocampus, we found that prenatal LPS exposure increases the activity of astrocyte connexin 43 (Cx43) hemichannels and pannexin-1 (Panx1) channels *ex vivo* in acute brain slices from adult offspring mice. Relevantly, the opening of Cx43 hemichannels affected the release of glutamate, [Ca^2+^]_i_ handling, and morphology of astrocytes, whereas also impaired the dendritic arbor and spine density, as well as neuronal survival.

## Materials and Methods

### Reagents and Antibodies

The mimetic peptides gap19 (KQIEIKKFK, intracellular loop domain of Cx43), gap19^I130A^ (KQAEIKKFK, negative control), Tat-gap19 (YGRKKRRQRRR-KQIEIKKFK, intracellular loop domain of Cx43), Tat-gap19^I130A^ (YGRKKRRQRRR-KQAEIKK FK, negative control), Tat-L2 (YGRKKRRQRRR-DGANVDM HLKQIEIKKFKYGIEEHGK, second intracellular loop domain of Cx43), Tat-L2^H126K/I130N^ (YGRKKRRQRRR-DGANVD MKLKQNEIKKFKYGIEEHGK, negative control), ^10^panx1 (WRQAAFVDSY, first extracellular loop domain of Panx1) and ^10^panx1^src^ (FSVYWAQADR, scrambled peptide) were obtained from Genscript (New Jersey, United States). HEPES, water (W3500), minocycline, SB203580, polyclonal anti-Cx43 antibody, anti-glial fibrillary acidic protein (GFAP) monoclonal antibody, minocycline, oATP, ethidium (Etd) bromide (Ex-Max 493 nm/Em-Max 620 nm), sulforhodamine 101 (SR101) (Ex-Max 586 nm/Em-Max 605 nm) and probenecid (Prob) were purchased from Sigma-Aldrich (St. Louis, MO, United States). A740003, U-73122, 2-APB, MTEP, SIB-1757, LN-6, and A740003 were obtained from Tocris (Bristol, United Kingdom). Fluo-4-AM (Ex-Max 494 nm/Em-Max 506 nm), DAF-FM diacetate (Ex-Max 495 nm/Em-Max 515 nm), monoclonal anti-Iba-1 antibody, BAPTA-AM, diamidino-2-phenylindole (DAPI) (Ex-Max 359 nm/Em-Max 461 nm), goat anti-mouse Alexa Fluor 488 (Ex-Max 495 nm/Em-Max 519 nm) were obtained from Thermofisher (Waltham, MA, United States). Fluoro-Jade C (F-Jade) (Ex-Max 485 nm/Em-Max 525 nm) were obtained from Chemicon (Martinsried/Munich, Germany). A soluble form of the TNF-α receptor (sTNF-αR1) and a recombinant receptor antagonist for IL-1β (IL-1ra) were from R&D Systems (Minneapolis, MN, United States).

### Animals

The animals were treated and handled according to the National Institutes of Health guidelines (NIH, Baltimore, MD, United States). The experimental procedures were approved by the Bioethical and Biosafety Committee of the Faculty of Biological Sciences at the Pontificia Universidad Católica de Chile. Mice of 8–9 weeks of age were housed in cages in a temperature-controlled (24°C) and humidity-controlled vivarium under a 12 h light/dark cycle (lights on 8:00 a.m.), with *ad libitum* access to food and water. The experiments performed in this study involved the following number of offspring animals per group of treatment: control (54), prenatal LPS (78), prenatal LPS + Tat-gap19 (6) and prenatal LPS + Tat-gap19^I130A^ (6) (see below for details).

### Prenatal LPS Exposure Protocol and Mimetic Peptide *in vivo* Administration

The protocol of inflammatory stimulation was applied on gestation day 17. Pregnant mice were randomly assigned to one of two groups: (1) control (0.9% NaCl, i.p. injection) and (2) prenatal LPS (0.01 μg/g *E. Coli* LPS, i.p. injection). Given that prenatal LPS inflammation may induce sex-specific brain effects in the offspring (Makinson et al., 2017), we used only male offspring in our studies. Following full term delivery male offspring were used to prepare acute brain slices. In some *in vivo* experiments, we used the gap19 mimetic peptide coupled to the TAT membrane translocation motif (Tat-gap19), which is known to cross the blood-brain barrier (Abudara et al., 2014). Although gap19 contains the KKFK sequence that is a known cell-membrane translocation motif that facilitates plasma membrane permeability (Carrigan and Imperiali, 2005), we used the TAT version of this peptide in order to increase its cell membrane permeability and chances to interact with its union site: the intracellular C-terminal tail of Cx43 (Abudara et al., 2014). An I130A-modified gap19 analog (Tat-gap19^I130A^) was employed as a negative control peptide because amino acid I130 is implicated in the formation of hydrogen bonds and thereby crucial for gap19 activity (Wang et al., 2013). Accordingly, Tat-Gap19^I130A^ exerts no inhibitory effects on Cx43 hemichannels (Wang et al., 2013). Tat-gap19 (23 mg/kg), Tat-gap19^I130A^ (23 mg/kg) or saline solution were administrated via intraperitoneal (i.p.) injections beginning on PND 30, as has been previously described to be useful in acute and long-lasting administration in rodents (Crespo Yanguas et al., 2018; Chen et al., 2019; Maatouk et al., 2019). A second dose was given on PND45 followed by injections in PND 60, 75, 90, and 105.

### Acute Brain Slices

Mice were anesthetized under isoflurane, decapitated and brains were extracted and cut into coronal slices (300 μm) using a vibratome (Leica, VT1000GS; Leica, Wetzlar, Germany) filled with ice-cold slicing solution containing (in mM): sucrose (222); KCl (2.6); NaHCO_3_ (27); NaHPO_4_ (1.5); glucose (10); MgSO_4_ (7); CaCl_2_ (0.5) and ascorbate (0.1), bubbled with 95% O_2_/5% CO_2__,_ pH 7.4. Then, the slices were transferred at room temperature (20–22°C) to a holding chamber in ice-cold artificial cerebral spinal fluid (ACSF) containing (in mM): NaCl (125), KCl (2.5), glucose (25), NaHCO_3_ (25), NaH_2_PO_4_ (1.25), CaCl_2_ (2), and MgCl_2_ (1), bubbled with 95% O_2_/5% CO_2_, pH 7.4, for a stabilization period of 60 min before dye uptake experiments (see below).

### Treatments

Some acute brain slices were pre-incubated for 15 min before and during experiments with the following agents: mimetic peptides against Cx43 hemichannels (Tat-L2 and gap19, 100 μM) and pannexin1 (Panx1) channels (^10^panx1, 100 μM), Prob (pannexin channel blocker, 500 μM), minocycline (inhibitor of microglial activation, 50 nM), sTNF-αR1 (soluble form of the receptor that binds TNF-α, 300 ng/ml), IL-1ra (IL-1β receptor endogenous blocker, 300 ng/ml), SB203580 (p38 MAP kinase inhibitor, 1 μM), L-N6 (iNOS inhibitor, 1 μM), A740003 (P2X_7_ receptor blocker, 200 nM), oATP (general P2X receptor blocker, 200 μM), BAPTA-AM (intracellular Ca^2+^ chelator, 10 μM), MTEP (selective mGluR_5_ antagonist, 50 nM), SIB-1757 (selective mGluR_5_ antagonist, 5 μM), U-73122 (selective phospholipase C (PLC) inhibitor, 5 μM), 2-APB (inhibitor of IP_3_ receptor antagonist, 50 μM), tetrodotoxin (TTX, 0. 5 μM).

### Dye Uptake in Acute Brain Slices and Confocal Microscopy

For dye uptake and *ex vivo* “snapshot” experiments, acute brain slices were incubated with 5 μM ethidium (Etd) for 10 min in a chamber filled with ACSF and bubbled with 95% O_2_/5% CO_2_, pH 7.4. Afterward, the slices were washed three times (5 min each) with ACSF, and fixed at room temperature with 4% paraformaldehyde for 60 min, rinsed once with 0.1 mM glycine in phosphate buffered saline (PBS) for 5 min and then twice with PBS for 10 min with gentle agitation. Then, the slices were incubated two times for 30 min each with a blocking solution (PBS, gelatin 0.2%, Triton-X 100 1%) at room temperature. Afterward, the slices were incubated overnight at 4°C with anti-GFAP monoclonal antibody (1:500, Sigma) to detect astrocytes. Additionally, some slices not previously subjected to Etd uptake were incubated overnight at 4°C with anti-Iba-1 monoclonal antibody (1:500, Thermofisher) to detect microglia or anti-polyclonal Cx43 antibody (1:400, SIGMA) to detect Cx43. Later, the slices were washed three times (10 min each) with blocking solution and then incubated for 2 h at room temperature with goat anti-mouse Alexa Fluor 488 (1:1000) antibody and Hoechst 33342. Further, the slices were washed three times (10 min each) in PBS and then mounted in Fluoromount, cover-slipped and examined in a confocal laser-scanning microscope (Eclipse Ti-E C2, Nikon, Japan). Stacks of consecutive confocal images were taken with 40X objective at 100 nm intervals were acquired sequentially with three lasers (in nm: 408, 488, and 543), and Z projections were reconstructed using Nikon confocal software (NIS-elements) and ImageJ software. At least six cells per field were selected from at least three fields in each brain slice. To assess the fluorescent intensity and distribution of Cx43 in astrocytes, stacks of consecutive confocal images were taken with the same confocal microscope, but with a 60X oil immersion objective (1.4 NA) at 200 nm intervals. Images were acquired sequentially with three lasers (in nm: 488 and 543), and Z projections were reconstructed using Nikon confocal software (NIS-elements). Image analysis of Z projections was then performed with ImageJ software. Cx43 intensity in areas close to the plasma membrane and cytoplasm was modeled by using the Otsu plugin for automatic image thresholding and the “enlarge” function of ImageJ. With the latter, we created a 10-pixel extension from the contour of the intracellular GFAP signal of each selected astrocyte to obtain an approximation of the plasma membrane area. Dye uptake or Cx43 fluorescence was calculated with the following formula: Corrected fluorescence = Integrated Density – ([Area of selected cell] x [Mean fluorescence of background readings]).

### Enzyme-Linked Immunosorbent Assay (ELISA)

ELISA assays were performed to determine the amount of TNF-α and IL-1β in the hippocampus. Mice were anesthetized with ketamine/xylazine (10:1 mg/kg of body weight, i.p.) and then perfused and decapitated. Afterward the hippocampus was removed and homogenized with an Ultra-Turrax homogenizer in buffer containing Tris-HCl 100 mM pH 7.4, EDTA 5 mM, SDS 1%, PMSF 1 μM and the protease inhibitor cocktail (ratio: 0.1 g hippocampus tissue: 1 ml lysis buffer) (Pierce, Rockford, IL, United States). Protein concentrations were determined by using a detergent-compatible Bio-Rad protein assay kit (Bio-Rad, Richmond, CA, United States). Then, the samples were centrifuged at 14,000 g for 10 min. Supernatants were collected and protein content assayed by BCA method. Cytokine levels were determined by sandwich ELISA, according to the manufacturer’s protocol (IL-1β and TNF-α EIA kit, Enzo Life Science, United States). For the assay, 100 μl of samples were added per ELISA plate well and incubated 4°C overnight. A calibration curve with recombinant cytokine was included. Detection antibody was incubated at room temperature for 2 h and the reaction developed with avidin–HRP and substrate solution. Absorbance was measured at 450 nm with reference to 570 nm with the microplate reader Synergy HT (Biotek Instruments). The results were normalized by protein amount.

### [Ca^2+^]_i_ and NO Imaging

Acute brain slices were incubated for 20 min at 34°C in ACSF solution containing 1 μM SR101, washed and processed for Fluo-4 AM (Ca^2+^ indicator) or DAF-FM (NO indicator) loading. For that, acute slices were incubated for 60 min at 37°C in ACSF containing 0.02% Pluronic F-127 and 5 μM Fluo-4 AM or 5 μM DAF-FM. Then, slices were transferred on the stage of a confocal laser scanning microscope and Ca^2+^ or NO measurements were carried out for 20 min. Fluo-4 or DAF-FM were excited with an argon laser (488 nm) and emission was filtered with a 515 ± 15 nm filter, whereas SR101 was excited with a HeNe green laser (543 nm) and emission was filtered with a 605 ± 75 nm filter. Acquisitions were carried out in the frame-scanning mode at 1 frame every 2 s with a 60x objective (NA 0.95; Nikon, Tokyo, Japan) on an Eclipse microscope (Nikon Instruments, Tokyo, Japan) equipped with and confocal head (confocal C2 head, Nikon) and controlled by the NIS-element software. The NO/Ca^2+^ imaging data was analyzed using FIJI-IMAGE-J programs. Images with obvious motion were excluded for analysis. ROIs in astrocytes, including somata and processes, were manually identified on the basis of morphology. Fluorescence intensity was calculated with the following formula: Corrected total cell fluorescence = Integrated Density – ([Area of selected cell] × [Mean fluorescence of background readings]). At least four cells per field were selected from at least three fields in each brain slice. For spontaneous [Ca^2+^]_i_ oscillations, the peaks were detected using the algorithm developed by Igor Pro from WaveMetrics. The frequency and amplitude were calculated and measured.

### Measurement of Extracellular ATP and Glutamate Concentration

Acute hippocampal slices were immersed in oxygenated ACSF (as above), pH 7.4, at room temperature (20–22°C) for 30 min either under control conditions or exposed to different agents. Then, extracellular ATP was measured using a luciferin/luciferase bioluminescence assay kit (Sigma-Aldrich, St. Louis, MO, United States), while extracellular levels of glutamate were determined using an enzyme-linked fluorimetric assay (Sigma-Aldrich, St. Louis, MO, United States). The amount of glutamate and ATP in each sample was inferred from standard curves. Briefly, after the experiments, the slices were washed twice with ACSF solution and sonicated in ice-cold PBS containing 5 μM EDTA, Halt (78440) and T-PER protein extraction cocktail (78510) according to manufacturer instructions (Pierce, Rockford, IL, United States). Total proteins from tissue homogenates were measured using the Bio-Rad protein assay.

### Golgi Staining

Mice were deeply anesthetized with isoflurane (4%) before euthanizing by decapitation. Brains were removed quickly from the skull to avoid any damage to the tissue. After rinsing, the tissue was sliced in approximately 10 mm thick blocks. The blocks were stained with the FD Rapid GolgiStain^TM^ kit (FD NeuroTechnologies, Ellicott City, MD, United States). They were first immersed in the impregnation solution (A and B) which was replaced after 6–12 h and then kept in dark for 15–16 days. Afterward, the blocks were put in Solution C which was replaced after 24 h and kept in dark for the next 48–60 h. Cryomicrotome (Microm Thermo Scientific, Walldorf, Germany) was used to cut 200 μm thick slices. Slices were mounted on a gelatin-coated microscope slides, stained, and dehydrated and coverslipped with Permount. Tissue preparation and staining were all done by the same person following the FD Rapid GolgiStain^TM^ kit manufacturer’s protocol. Neuronal dendritic arbors and spines were imaged using motorized microscope-computer based system and the MFB Stereo Investigator software version 11 (MBF–Bioscience, Williston, ND, United States). System was composed of z-axis motorized Olympus BX51 microscope equipped with x-y motorized stage guided by MAC5000 stage controller (Ludl Electronic Products Ltd., Hawthorne, NY, United States).

### Morphometry and Sholl Analysis

Image processing of slices labeled for microglia (Iba-1 immunostaining), astrocytes (GFAP immunostaining) or neurons (golgi staining) was performed using the Fiji-ImageJ software (Schindelin et al., 2012). All samples were coded and analyzed randomly by a researcher blinded to animal number and condition. A minimum of 10 cells from each animal where chosen for analysis and their image data were imported using the BioFormats plugin and then channels separated with the Split channels tool. Later, the Iba-1, GFAP or golgi channel were selected and Z-axis projection of the sum of planes was performed using the Z projection tool. Afterward, microglia, astrocytes or neurons were selected and cut with the crop tool to facilitate their analysis when they fulfilled the following criteria: (i) presence of untruncated processes, (ii) consistent and strong staining along the entire arborization field, and (iii) relative isolation from neighboring cells to avoid overlap. Afterward, signal was segmented with the threshold tool and converted to binary mask before its skeletonization with the skeletonize tool. The latter tool allowed to obtain segment length and any possible bifurcation of the skeletonized image analyzed with the Fiji-ImageJ software. Due their complexity, drawings of neurons were done before skeletonization by using the Neuromantic software (Myatt et al., 2012), which allow the semi-manual or semi-automatic reconstruction of neurons from single images or image stacks. Then various features were measured including maximum and total branch length of cell processes, number of terminals, maximum path distance (maximum length of a path between the soma and terminal dendrites), as well as the number of branches were measured with the AnalizeSkeleton plugin of Fiji-ImageJ and/or the Neuromantic software. Further, the plugin Sholl analysis of Fiji-ImageJ was used to place concentric circles around the cell starting from the soma and radiating outward at increasing radial increments of 5 μm (Sholl, 1953). Different parameters were measured including the numbers of intersections (points where the cellular processes cross concentric rings), area under the Sholl curve, the maximum number of intersections, the radius of highest count of intersections (maximum intersect. radius) and the sum of intersections divided by intersecting radii (mean of intersections).

### Spine Density Estimation

Dendrite spine counting was conducted blind to the experimental condition. Measurements were obtained from the CA1 area of the dorsal hippocampus, whereas secondary or tertiary dendrite branches from the apical part (stratum radiatum) and from the basal part (stratum oriens) of the pyramidal cells were analyzed. Dendrite fragments chosen for analysis had to meet the following criteria: (i) good staining and impregnation without breaks, (ii) location about 150 μm (apical part) or about 40 μm (basal part) from the soma, (ii) branch fragments must be in the same focus plane and have a length about 30 μm (20–50 μm), and (iv) the branch fragment must be relatively straight to minimize errors connected with length measuring. About 8 fragments per brain were analyzed and spines were counted in the Fiji-ImageJ software. Afterward, signal was segmented with the threshold tool and converted to binary mask before its skeletonization with the skeletonize tool. The latter tool allowed to obtain segment length and the number of spines using the semiautomatic counting plugin of the Fiji-ImageJ software.

### Neuronal Death Quantification

Acute brain slices were fixed in 40% ethanol at 4°C for 5 min, treated with 0.1% Triton X-100 in PBS for 10 min and rinsed twice with distilled water. Preparations were incubated with 0.001% F-Jade in distilled water and gently shaken for 30 min in the dark. Later, F-Jade was removed and slices were incubated with anti-GFAP monoclonal antibody (Sigma, 1:400) diluted in 0.1% PBS-Triton X-100 with 2% NGS at 4°C overnight. After five rinses in 0.1% PBS-Triton X-100, slices were incubated with goat anti-mouse IgG Alexa Fluor 488 (1:1000) at room temperature for 50 min. After several washes, coverslips were mounted in DAKO fluorescent mounting medium and examined with a confocal laser-scanning microscope (Olympus, Fluoview FV1000, Tokyo, Japan).

### Statistical Analysis

For each data group, results were expressed as mean ± standard error (SEM); n refers to the number of independent experiments. Detailed statistical results were included in the figure legends. Statistical analyses were performed using GraphPad Prism (version 7, GraphPad Software, La Jolla, CA, United States). Normality and equal variances were assessed by the Shapiro–Wilk normality test and Brown–Forsythe test, respectively. Unless otherwise stated, data that passed these tests were analyzed by unpaired *t*-test in case of comparing two groups, whereas in case of multiple comparisons, data were analyzed by one or two-way analysis of variance (ANOVA) followed, in case of significance, by a Tukey’s *post hoc* test. When data were heteroscedastic as well as not normal and with unequal variances, we used Mann–Whitney test in case of comparing two groups, whereas in case of multiple comparisons data are analyzed by Kruskal–Wallis test followed, in case of significance, by Dunn’s *post hoc* test. A probability of *P* < 0.05 was considered statistically significant.

## Results

### Prenatal LPS Exposure Enhances the Opening of Cx43 Hemichannels and Panx1 Channels in the Hippocampus of Adult Offspring Mice

The offspring of LPS-exposed dams exhibit alterations in hippocampal-dependent synaptic plasticity and memory (Hao et al., 2010; Kelley et al., 2017), as well as increased neuronal death and astrogliosis (Ling et al., 2002; Zager et al., 2015). Because the exacerbated activity of astrocyte hemichannels and pannexons impact synaptic impairment, neuronal loss and astrogliosis in the hippocampus (Abudara et al., 2015; Yi et al., 2016; Gomez et al., 2018), we examined whether prenatal LPS exposure modulates the functional activity of these channels in the hippocampal CA1 region of the offspring. For that reason, we investigated hemichannel and pannexon activity by measuring ethidium (Etd) uptake in acute brain slices from the offspring mice following different months after birth. Etd enters to the cytosol of activated cells through selective large-pore channels, including hemichannels and pannexons. After its intercalation with base pairs of DNA and RNA, Etd becomes fluorescent, denoting channel activity (Johnson et al., 2016). Etd uptake by GFAP-positive astrocytes on acute brain slices was studied taking “snapshot” images in the stratum radiatum of the hippocampal CA1 region.

**FIGURE 1:**
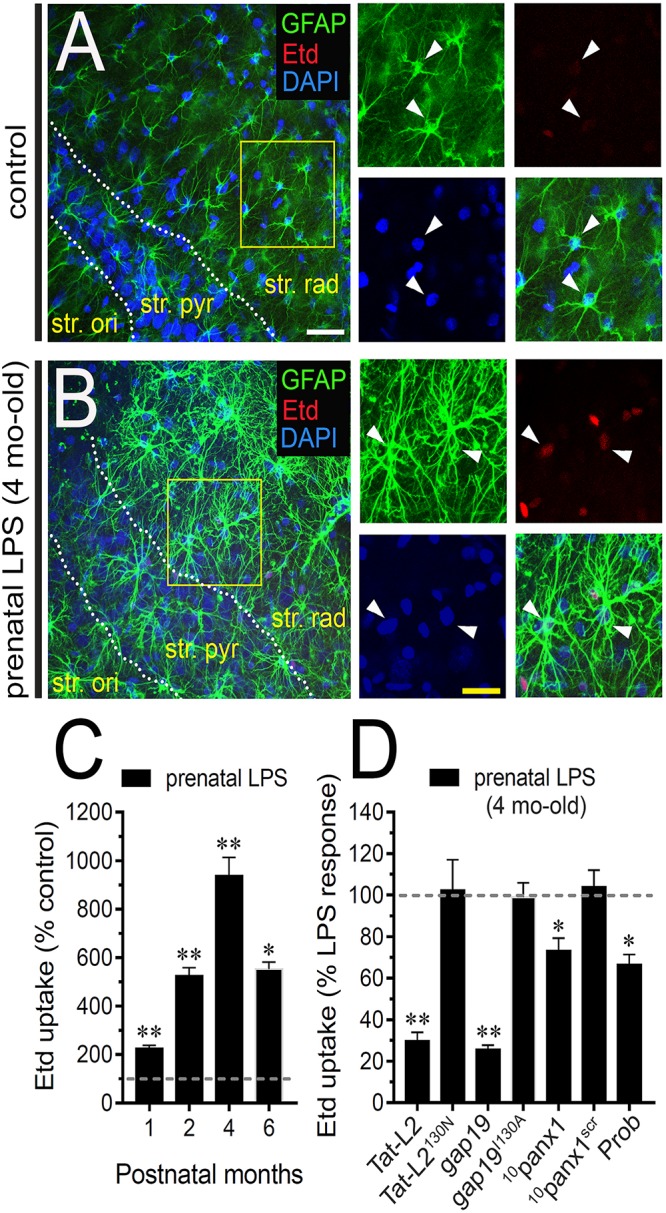
Prenatal LPS exposure augments the activity of Cx43 hemichannels and Panx1 channels by astrocytes on offspring hippocampus. Representative images showing GFAP (green), Etd (red) and DAPI (blue) staining in the hippocampus of control offspring **(A)** or prenatally LPS-exposed offspring **(B)** of 4 months old. Insets of astrocytes were taken from the area depicted within the yellow squares in **(A,B)**. **(C)** Averaged data of Etd uptake normalized to control conditions (dashed line) by hippocampal astrocytes in acute slices from prenatally LPS-exposed offspring after following different postnatal periods. ^∗∗^*p* < 0.0001, ^∗^*p* < 0.001 versus control, one-way ANOVA Tukey’s *post hoc* test, mean ± S.E.M., *n* = 3. **(D)** Averaged data normalized to the maximal effect (dashed line) induced by prenatal LPS exposure on Etd uptake by hippocampal astrocytes in acute slices from 4 months old offspring exposed to the following pharmacological agents: 100 μM Tat-L2, 100 μM Tat-L2^H126K/I130N^, 100 μM gap19, 100 μM gap19^I130A^, 100 μM ^10^panx1, 100 μM ^10^panx1^scrb^ and 500 μM Probenecid (Prob). ^∗∗^*p* < 0.0001, ^∗^*p* < 0.05 versus LPS, one-way ANOVA Tukey’s *post hoc* test, mean ± S.E.M., *n* = 3. Calibration bars: white bar = 180 μm; yellow bar: 100 μm.

Astrocytes analyzed in acute brain slices from control offspring displayed a weak Etd uptake in the stratum radiatum (Figure 1A). Nonetheless, 4 months old offspring mice from LPS-exposed dams exhibited hippocampal astrocytes with increased Etd uptake compared to control conditions (∼900%, Figures 1A–C). Temporal analysis of these responses showed that astroglial Etd uptake rapidly increased 1 month after birth in prenatally LPS-exposed offspring and reached a maximum in 4 months old offspring (Figure 1C). Thus, hereinafter and unless otherwise stated, this postnatal period was used in all further experiments throughout this study. Because Cx43 hemichannels and Panx1 channels are pivotal pathways for dye passage in astrocytes (Contreras et al., 2002; Iglesias et al., 2009), the potential involvement of these channels in the prenatal LPS-induced astroglial Etd uptake was investigated. Accordingly, acute brain slices were pre-incubated for 15 min before and during Etd uptake recordings with a battery of diverse pharmacological molecules. Tat-L2 (100 μM) and gap19 (100 μM); two mimetic peptides that inhibit Cx43 hemichannels by biding the intracellular L2 loop of Cx43 (Iyyathurai et al., 2013); strongly blunted the prenatal LPS-evoked Etd uptake in hippocampal astrocytes to ∼30 and 26% compared to 100% of the maximum response, respectively (Figure 1D). Moreover, an adapted Tat-L2 (Tat-L2^H126K/I130N^), in which 2 aa essential for binding of L2 to the CT tail of Cx43 are mutated, did not cause an equivalent inhibitory response (Figure 1D). Equivalently, we noted that an inactive structure of gap19 containing the I130A modification (gap19^I130A^), failed to reduce the prenatal LPS-dependent Etd uptake in astrocytes (Figure 1D). To elucidate the participation of Panx1 channels to the prenatal LPS-induced Etd uptake in hippocampal astrocytes, we employed the mimetic peptide ^10^panx1 with an amino acid sequence homologous to the first extracellular loop domain of Panx1 (Pelegrin and Surprenant, 2006), as well as probenecid. ^10^panx1 (100 μM) and probenecid (500 μM) but not a scrambled peptide for ^10^panx1 partially counteracted the prenatal LPS-mediated astrocyte Etd uptake (Figure 1D). Collectively, this evidence suggests that prenatal LPS exposure augments the opening of astrocyte Cx43 hemichannels and Panx1 channels in the hippocampus from the adult offspring.

### Activation of Microglia and IL-1β/TNF-α/p38 MAP Kinase/iNOS Signaling Contribute to the Opening of Astrocyte Cx43 Hemichannels in Prenatally LPS-Exposed Adult Offspring

**FIGURE 2:**
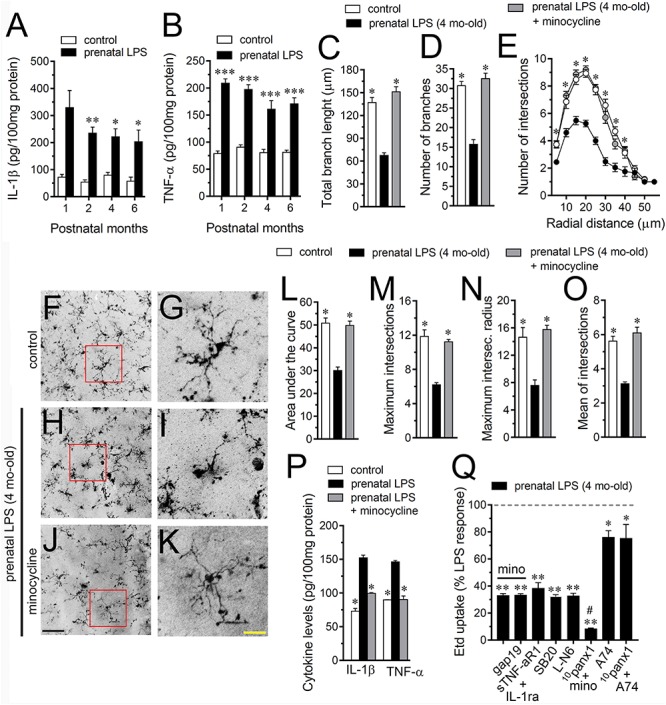
Microglia and IL-1β/TNF-α/p38 MAP kinase/iNOS signaling participate in the prenatal LPS-induced opening of astrocyte Cx43 hemichannels on offspring hippocampus. Averaged data of hippocampal levels of IL-1β **(A)** and TNF-α **(B)** from control offspring (white bars) or prenatally LPS-exposed offspring (black bars) following different postnatal periods. ^∗∗∗^*p* < 0.0001, ^∗∗^*p* < 0.005, ^∗^*p* < 0.05 versus control, two-way ANOVA Bonferroni’s *post hoc* test, mean ± S.E.M., *n* = 3. **(C,D)** Averaged data of total branch length **(C)** and number of branches **(D)** by hippocampal microglia in acute slices from control offspring (white bars) or prenatally LPS-exposed offspring (black bars) of 4 months old. Also shown are the effects of treatment with 50 nM minocycline for 2 h in acute slices prenatally LPS-exposed offspring of 4 months old (gray bars). ^∗^*p* < 0.0001 versus LPS, one-way ANOVA Dunnett’s *post hoc* test, mean ± S.E.M., *n* = 3. **(E)** Averaged data of Sholl analysis by hippocampal microglia from control offspring (white circles) or prenatally LPS-exposed offspring of 4 months old alone (black circles) or plus treatment with 50 nM minocycline (gray circles). ^∗^*p* < 0.001 versus LPS, two-way ANOVA Tukey’s *post hoc* test, mean ± S.E.M., *n* = 3. **(F–K)** Representative Iba-1 (black) positive hippocampal microglia in acute slices from control offspring **(F,G)** or prenatally LPS-exposed offspring of 4 months old alone **(H,I)** or plus treatment with 50 nM minocycline **(J,K)**. Insets of microglia **(G,I,K)** were taken from the area depicted within the red squares in **(F,H,J)**. **(L–O)** Averaged data of area under the curve of Sholl analysis **(L)**, maximum intersection **(M)**, maximum intersection radius **(N)**, and mean of intersections **(O)** by hippocampal microglia from control offspring (white bars) or prenatally LPS-exposed offspring of 4 months old alone (black bars) or plus treatment with 50 nM minocycline (gray bars). ^∗^*p* < 0.0001 versus LPS, one-way ANOVA Dunnett’s *post hoc* test, mean ± S.E.M., *n* = 3. **(P)** Averaged data of hippocampal levels of IL-1β and TNF-α from control offspring (white bars) or prenatally LPS-exposed offspring of 4 months old alone (black bars) or plus treatment with 50 nM minocycline (gray bars). ^∗∗∗^*p* < 0.0001, versus control, two-way ANOVA Bonferroni’s *post hoc* test, mean ± S.E.M., *n* = 3. **(Q)** Averaged data normalized to the maximal effect (dashed line) induced by prenatal LPS exposure on Etd uptake by hippocampal astrocytes in acute slices from 4 months old offspring exposed to the following pharmacological agents: 50 nM minocycline, 50 nM minocycline + 100 μM gap19, sTNF-αR1 + IL-1ra (300 ng/ml each), 1 μM SB203580, 1 μM L-N6, 50 nM minocycline + 100 μM ^10^panx1, 200 nM A740003 or 100 μM ^10^panx1 + 200 nM A740003. ^∗∗^*p* < 0.0001, ^∗^*p* < 0.005 versus LPS, ^#^*p* < 0.0001 versus minocycline, one-way ANOVA Dunnett’s *post hoc* test, mean ± S.E.M., *n* = 3. Calibration bars: black bar = 180 μm; yellow bar: 80 μm.

Given that release of inflammatory cytokines is critical for modulating molecular, morphological and functional properties of astrocytes during pathological conditions (Agulhon et al., 2012), we evaluated whether prenatal LPS exposure could modulate the hippocampal levels of these cytokines in the offspring. During the 1 month period after birth, the hippocampus of prenatally LPS-exposed offspring showed a strong ∼5.5-fold increase in IL-1β levels compared to control that then dropped progressively in the following months (Figure 2A). Likewise, prenatal LPS exposure triggered a prominent 2.5-fold increase in hippocampal TNF-α levels of 1 month old offspring, which was slightly decreasing over time (Figure 2B). One of the major sources of cytokine production in the brain is the microglia and its activation has been observed along with neuroinflammation in prenatally LPS-exposed offspring (Schaafsma et al., 2017). Given that activation of microglia occurs along with changes in their morphology (Kettenmann et al., 2011), we measured the total branch length and branch points of microglial processes at the stratum radiatum. Analysis starting at the cell body throughout the end of each process, permit us to calculate the sum of all branch lengths and number of branch points of each microglia arbor. We found that prenatal LPS exposure reduced the branch points and the total length of microglial processes in the 4 months old offspring hippocampus (Figures 2C,D). Relevantly, microglia processes from prenatally LPS-exposed offspring showed similar branch points and length than their control counterparts when brain slices were treated with 50 nM minocycline (Figures 2C,D), a molecule that attenuates microglial activation (Kim and Suh, 2009).

To explore deeper the arbor complexity of microglia in the prenatally LPS-exposed offspring, we employed a Sholl analysis, which consists in place concentric rings at established intervals from the soma to then count branch intersections at each ring. We observed that hippocampal microglia of the offspring of LPS-exposed dams are significantly different from those of control offspring (Figures 2E–K). Particularly, during 4 months after birth, a dramatical reduction in the number of intersections between branches and Sholl rings was detected in hippocampal microglia of prenatally LPS-exposed offspring (Figure 2E). Prenatal LPS exposure also reduced microglial branch complexity as measured by the area under the Sholl curve for the total number of branch intersections at 5–60 μm from the soma (Figure 2L). Furthermore, hippocampal microglia of the offspring of LPS-exposed dams also exhibited decreased values in the maximum number of intersections, the radius of highest count of intersections (maximum intersect. radius) and the sum of intersections divided by intersecting radii (mean of intersections) (Figures 2M–O). Relevantly, minocycline treatment greatly prevented not only the arbor reduction and altered morphology observed in hippocampal microglia from prenatally LPS-exposed offspring (Figures 2E–O), but also the increased production of IL-1β and TNF-α occurring in these conditions (Figure 2P).

On the other hand, we found that minocycline prominently blunted the prenatal LPS-induced Etd uptake in hippocampal astrocytes, whereas pretreatment with a soluble form of TNF-α receptor that binds TNF-α (sTNF-aR1) and a recombinant antagonist for IL-1β receptor (IL-1ra) caused equivalent responses (Figure 2Q). It is known that IL-1β and TNF-α along with p38 MAP kinase activation, lead to NO-dependent S-nitrosylation of astrocytic Cx43 hemichannels, increasing their activity (Retamal et al., 2007). In this line, we found that the prenatal LPS-induced Etd uptake in hippocampal astrocytes was prominently tackled by blocking p38 MAP kinase with 10 μM SB202190 or of iNOS by 5 μM L-N6 (Figure 2Q). Altogether, these observations reveal that TNF-α/IL-1β and activation of iNOS/p38 MAP kinase pathways appear to be pivotal for the prenatal LPS-evoked opening of astrocyte Cx43 hemichannels but not Panx1 channels in the hippocampus. Accordingly, the Cx43 hemichannel blocker gap19 failed to trigger any additive inhibition in the prenatal LPS-induced Etd uptake when slices were treated with minocycline (Figure 2Q). By contrast, the Panx1 channel blocker ^10^panx1 caused an additive inhibition when slices were stimulated with minocycline (Figure 2Q), suggesting that pannexon activity is not linked to the release of cytokines from activated microglia.

Prior studies have described that opening of Panx1 channels relies on direct protein-protein interactions with P2X_7_ receptors (P2X_7_Rs) (Iglesias et al., 2008). According with this evidence, we found that 200 nM A740003, a selective P2X_7_R antagonist, caused a partial reduction in the prenatal LPS-induced Etd uptake in hippocampal astrocytes (Figure 2Q), which was close to the inhibitory effect induced by Panx1 channel blockers (Figure 1D). Relevantly, ^10^panx1 did not evoke any additive inhibition on Etd uptake of that caused by A740003 (Figure 2Q), underscoring the possibility that prenatal LPS-induced opening of Panx1 channels could take place via the activation of P2X_7_Rs.

### Prenatal LPS Exposure Increases the Astrocyte Production of NO and the Release of ATP via Panx1 Channels in the Offspring Hippocampus

**FIGURE 3:**
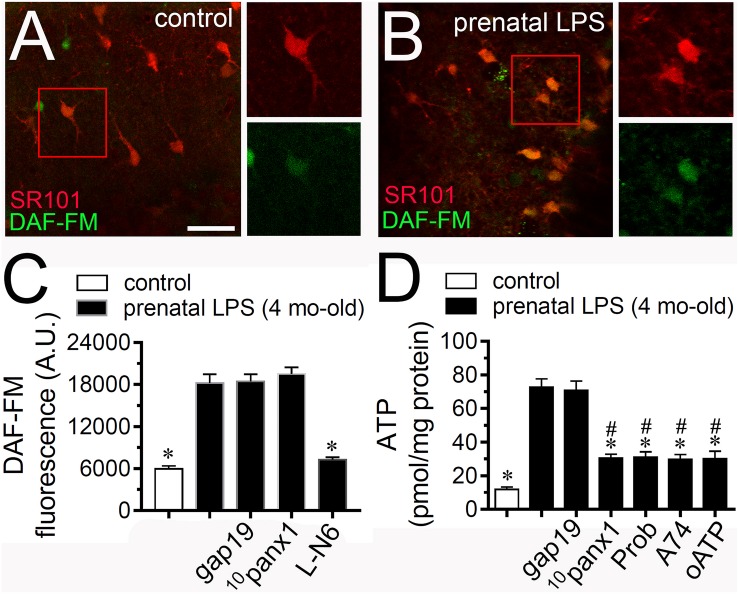
Prenatal LPS exposure increases the production of NO by astrocytes and the Panx1 channel-dependent release of ATP on offspring hippocampus. Representative images showing SR101 (red) and DAF-FM (green) staining by hippocampal astrocytes in acute slices from control offspring **(A)** or prenatally LPS-exposed offspring **(B)** of 4 months old. Insets of astrocytes were taken from the area depicted within the red squares in **(A,B)**. **(C)** Averaged of DAF-FM signal fluorescence by astrocytes in acute slices from control offspring (white bar) or prenatally LPS-exposed offspring of 4 months old alone (black bars) or in combination with the following pharmacological agents: 100 μM gap19, 100 μM ^10^panx1 and 1 μM L-N6. ^∗^*p* < 0.0001 versus LPS, one-way ANOVA Tukey’s *post hoc* test, mean ± S.E.M., *n* = 3. **(D)** Averaged data of ATP release by acute hippocampal slices from control offspring (white bar) or prenatally LPS-exposed offspring of 4 months old alone (black bars) or in combination with the following blockers: 100 μM gap19, 100 μM ^10^panx1 and 1 μM L-N6. ^∗^*p* < 0.0001 versus LPS, ^#^*p* < 0.0001 versus control, one-way ANOVA Tukey’s *post hoc* test, mean ± S.E.M., *n* = 3. Calibration bar = 85 μm.

Given that NO opens Cx43 hemichannels (Retamal et al., 2006) and because inhibition of iNOS with LN-6 greatly reduced the Etd uptake caused by prenatal LPS exposure in hippocampal astrocytes (Figure 2Q), we tested if this condition could affect NO production in the offspring hippocampus. DAF-FM (NO indicator) and SR101 (astrocyte marker) fluorescence imaging revealed that hippocampal astrocytes from prenatally LPS-exposed offspring showed a ∼2.5-fold augment in basal NO production compared to control conditions (Figures 3A–C). The fact that LN-6 totally suppressed the prenatal-LPS-induced production of NO indicates that iNOS is the major contributor to this response (Figure 3C). A previous study has related the opening of Panx1 channels with the production of NO (Orellana et al., 2013). In opposition to this finding, we observed that neither ^10^panx1 nor gap19 were effective in to prevent the prenatal-LPS-induced production of NO (Figure 3C), suggesting that Panx1 channels and Cx43 hemichannels and are not involved in this response.

iNOS stimulation is crucial for the hemichannel/pannexon-dependent release of ATP from astrocytes occurring after LPS treatment (Avendano et al., 2015). With this in mind and given that A740003, a selective antagonist of P2X_7_Rs, counteracted the astrocyte Etd uptake triggered by prenatal LPS exposure (Figure 2Q), we investigated if this condition could impact the release of ATP in the offspring hippocampus. Measurements of extracellular ATP levels with the luciferin/luciferase bioluminescence assay showed that prenatal LPS exposure dramatically augmented the release of ATP in ∼7-fold in the offspring hippocampus compared to control conditions (Figure 3D). Importantly, probenecid or ^10^panx1 but not gap19 markedly reduced the release of ATP caused by prenatal LPS exposure (from ∼73 pmol/mg to ∼30 pmol/mg and ∼31 pmol/mg, respectively) (Figure 3D). Similarly, 200 μM oATP or 200 nM A740003 prominently reduced the prenatal LPS-evoked release of ATP (Figure 3B).

### Cx43 Hemichannel Opening Evoked by Prenatal LPS Exposure Contributes to [Ca^2+^]_i_ and Glutamatergic Signaling on Offspring Hippocampus

**FIGURE 4:**
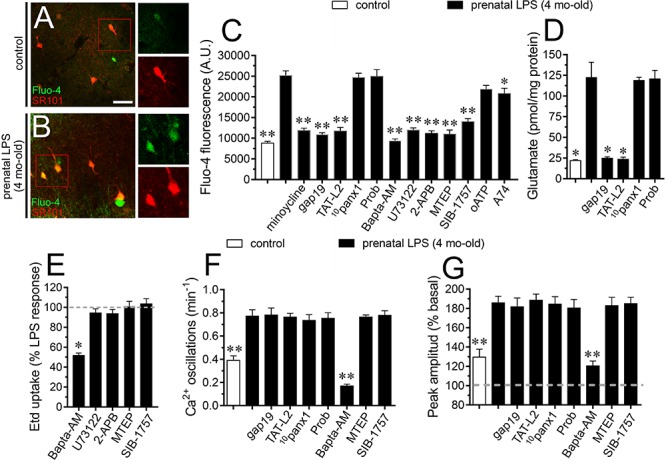
Prenatal LPS-induced opening of Cx43 hemichannels increases [Ca^2+^]_i_ and glutamatergic signaling on offspring hippocampus. Representative images showing SR101 (red) and Fluo-3 (green) staining by hippocampal astrocytes in acute slices from control offspring **(A)** or prenatally LPS-exposed offspring **(B)** of 4 months old. Insets of astrocytes were taken from the area depicted within the red squares in (A,B). **(C)** Averaged of basal Fluo-4 signal fluorescence by hippocampal astrocytes in acute slices from control offspring (white bar) or prenatally LPS-exposed offspring of 4 months old alone (black bars) or in combination with the following pharmacological agents: 50 nM minocycline, 100 μM gap19, 100 μM Tat-L2, 100 μM ^10^panx1, 500 μM Probenecid (Prob), 10 μM Bapta-AM, 5 μM U-73122, 50 μM 2-APB, 50 nM MTEP, 5 μM SIB-1757, 200 μM oATP and 200 nM A740003. ^∗∗^*p* < 0.0001, ^∗^*p* < 0.005 versus LPS, one-way ANOVA Tukey’s *post hoc* test, mean ± S.E.M., *n* = 3. **(D)** Averaged data of glutamate release by acute hippocampal slices from control offspring (white bar) or prenatally LPS-exposed offspring of 4 months old alone (black bars) or in combination with the following blockers: 100 μM gap19, 100 μM Tat-L2, 100 μM ^10^panx1, 500 μM Probenecid (Prob). ^∗^*p* < 0.0001 versus LPS, one-way ANOVA Tukey’s *post hoc* test, mean ± S.E.M., *n* = 3. **(E)** Averaged data normalized to the maximal effect (dashed line) induced by prenatal LPS exposure on Etd uptake by hippocampal astrocytes in acute slices from 4 months old offspring exposed to the following pharmacological agents: 10 μM Bapta-AM, 5 μM U-73122, 50 μM 2-APB, 50 nM MTEP, and 5 μM SIB-1757. ^∗^*p* < 0.0001 versus LPS, one-way ANOVA Tukey’s *post hoc* test, mean ± S.E.M., *n* = 3. **(F)** Averaged of spontaneous [Ca^2+^]_i_ oscillations by hippocampal astrocytes in acute slices from control offspring (white bar) or prenatally LPS-exposed offspring of 4 months old alone (black bars) or in combination with the following pharmacological agents: 100 μM gap19, 100 μM Tat-L2, 100 μM ^10^panx1, 500 μM Probenecid (Prob), 10 μM Bapta-AM, 50 nM MTEP or 5 μM SIB-1757. ^∗∗^*p* < 0.005 versus LPS, one-way ANOVA Tukey’s *post hoc* test, mean ± S.E.M., *n* = 3. **(G)** Averaged of peak amplitude of spontaneous [Ca^2+^]_i_ oscillations by hippocampal astrocytes in acute slices from control offspring (white bar) or prenatally LPS-exposed offspring of 4 months old alone (black bars) or in combination with the following pharmacological agents: 100 μM gap19, 100 μM Tat-L2, 100 μM ^10^panx1, 500 μM Probenecid (Prob), 10 μM Bapta-AM, 50 nM MTEP or 5 μM SIB-1757. ^∗∗^*p* < 0.005 versus LPS, one-way ANOVA Tukey’s *post hoc* test, mean ± S.E.M., *n* = 3. Calibration bar = 85 μm.

At the next step, we decided to analyze the effect of hemichannel/pannexon blockers on astroglial [Ca^2+^]_i_ in prenatally LPS-exposed offspring. As indicated by the assessment of Fluo-4 (Ca^2+^ indicator) and SR101, hippocampal astrocytes from offspring of LPS-exposed dams showed a 3-fold augment in basal levels of Ca^2+^ signal compared to control astrocytes (Figures 4A–C). Importantly, blockade of Cx43 hemichannels with Tat-L2 or gap19 dramatically suppressed the prenatal LPS-mediated increase in astroglial [Ca^2+^]_i_ in the hippocampus (Figure 4C). Similar observations were obtained upon treatment with minocycline but not with probenecid or ^10^panx1 (Figure 4C). Altogether these findings indicate that microglial-dependent opening of Cx43 hemichannels but not Panx1 channels participate in the prenatal LPS-induced increase in astroglial [Ca^2+^]_i_ in the hippocampus.

Glutamate modulates [Ca^2+^]_i_ dynamics in astrocytes, particularly through the stimulation of metabotropic glutamate receptors (mGluRs) and subsequent release of Ca^2+^ from intracellular stores (Bradley and Challiss, 2012). Here, we found that prenatal LPS-evoked [Ca^2+^]_i_ response was prominently blunted by 50 nM MTEP or 5 μM SIB-1757, being the latter two selective antagonists of mGluR_5_ (Figure 4C). Noteworthy, selective blockade of phospholipase C (PLC) or IP_3_ receptors with 5 μM U73122 or 50 μM APB, respectively, as well as chelation of [Ca^2+^]_i_ with 10 μM BAPTA-AM, strikingly counteracted the increase in astroglial [Ca^2+^]_i_ triggered by prenatal LPS exposure (Figure 4C). On the other hand, inhibition of P2X_7_Rs with oATP or A740003 caused a slight reduction in the prenatal LPS-induced astroglial [Ca^2+^]_i_ signal (Figure 4C). This could imply that astroglial [Ca^2+^]_i_ response resulting from prenatal LPS exposure is a consequence of the Cx43 hemichannel-dependent release of glutamate and further stimulation of mGluR_5_ rather than signaling via Panx1 channels. Consistent with this notion, we saw that prenatal LPS exposure induced a 7-fold increase in the release of glutamate in the offspring hippocampus, a response that was totally blunted by Tat-L2 or gap19 but not with probenecid or ^10^panx1 (Figure 4D). Further, we tested whether [Ca^2+^]_i_ and mGluR_5_ signaling were implicated in the Etd uptake observed in hippocampal astrocytes from prenatally LPS-exposed offspring. BAPTA-AM, but not inhibition of PLC, IP_3_ receptors or mGluR_5_, significantly reduced the prenatal LPS-induced Etd uptake in hippocampal astrocytes (Figure 4E). In this scenario, we further performed the analysis of spontaneous [Ca^2+^]_i_ oscillations in astrocytes. We found that prenatal LPS exposure increase the number of spontaneous astroglial [Ca^2+^]_i_ oscillations and their amplitude in the offspring hippocampus, a response that was totally blunted by BAPTA-AM but not by gap19, Tat-L2, ^10^panx1, probenecid, MTEP or SIB-1757 (Figure 4F). Similar responses were observed when the peak amplitude of spontaneous astroglial [Ca^2+^]_i_ oscillations was analyzed (Figure 4G). To figure out the possible contribution of neurons to these responses, we performed the above experiments in presence of 0.5 μM TTX. We found that TTX did not affect the prenatal LPS-induced changes in NO and [Ca^2+^]_i_ levels, as well as the release of glutamate, suggesting that neurons do not participate in these processes under these conditions (Supplementary Figures S1A–E). Collectively, these data suggest that spontaneous [Ca^2+^]_i_ oscillations evoked by prenatal LPS exposure are likely necessary for the opening of astroglialCx43 hemichannels, whereas the subsequent release of glutamate through them is needed for the increase in basal [Ca^2+^]_i_ via the activation of mGluR_5_ receptors. Although previous studies have associated the channel-dependent Etd uptake with changes in the distribution of Cx43 in astrocytes (Avendano et al., 2015), we found that prenatal LPS exposure did not alter the total amount or distribution of Cx43 in hippocampal astrocytes (Supplementary Figures S2A–G).

### Activation of Cx43 Hemichannels Contributes to Increased Arborization of Hippocampal Astrocytes in Prenatally LPS-Exposed Offspring

**FIGURE 5:**
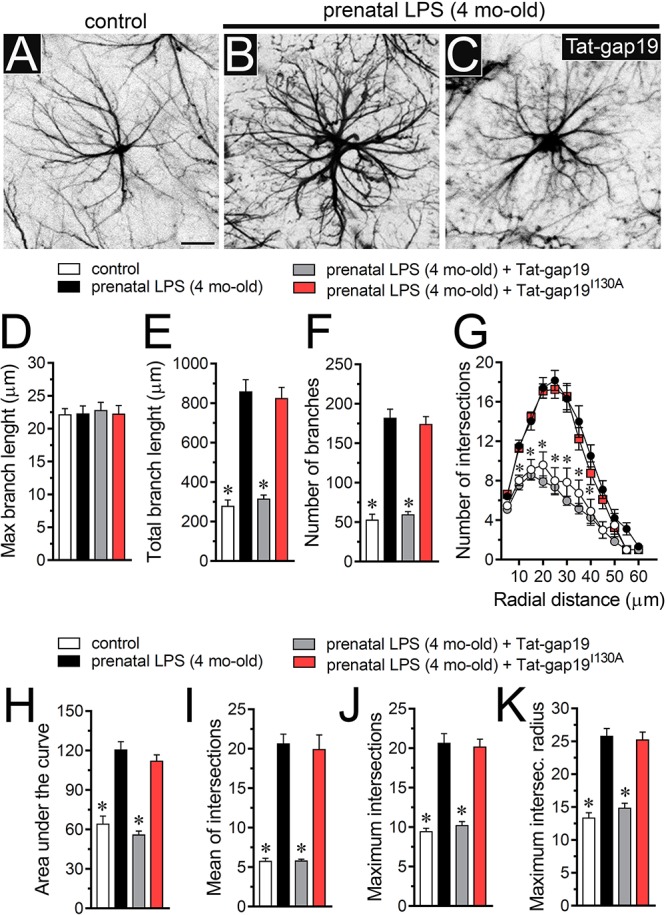
Prenatal LPS exposure increases the arborization of hippocampal astrocytes in the offspring by a mechanism involving the activation of Cx43 hemichannels. **(A–C)** Representative GFAP (black) positive hippocampal astrocytes from control offspring **(A)** or prenatally LPS-exposed offspring of 4 months old alone **(B)** or plus the *in vivo* administration of 23 mg/kg Tat-gap19 **(C)**. **(D–F)** Averaged data of maximum branch length **(D)**, total branch length **(E)** and number of branches **(F)** by hippocampal astrocytes in acute slices from control offspring (white bars) or prenatally LPS-exposed offspring (black bars) of 4 months old. Also shown are the effects of *in vivo* administration of 23 mg/kg Tat-gap19 (gray bars) or its inactive form: 23 mg/kg Tat-gap19^I130A^ (red bars). ^∗^*p* < 0.0001 versus LPS, one-way ANOVA Dunnett’s *post hoc* test, mean ± S.E.M., *n* = 3. **(G)** Averaged data of Sholl analysis by hippocampal astrocytes from control offspring (white circles) or prenatally LPS-exposed offspring of 4 months old alone (black circles) or plus the *in vivo* administration of 23 mg/kg Tat-gap19 (gray circles) or 23 mg/kg Tat-gap19^I130A^ (red circles). ^∗^*p* < 0.001 versus LPS, two-way ANOVA Tukey’s *post hoc* test, mean ± S.E.M., *n* = 3. **(H–K)** Averaged data of area under the curve of Sholl analysis **(H)**, maximum intersection **(I)**, maximum intersection radius **(J)**, and mean of intersections **(K)** by hippocampal astrocytes from control offspring (white bars) or prenatally LPS-exposed offspring of 4 months old alone (black bars) or plus the *in vivo* administration of 23 mg/kg Tat-gap19 (gray bars) or 23 mg/kg Tat-gap19^I130A^ (red bars). ^∗^*p* < 0.0001 versus LPS, one-way ANOVA Dunnett’s *post hoc* test, mean ± S.E.M., *n* = 3. Calibration bar = 40 μm.

One of the crucial aspects of reactive astrogliosis is the hypertrophy of cellular processes accompanied by crucial changes in the arborization and morphology of astrocytes (Pekny and Pekna, 2014). To understand whether the above described prenatal LPS-induced changes in astrocytes are accompanied by alterations in their arborization, we analyzed the maximum and total branch length of astroglial processes at the stratum radiatum (Figures 5A–E). Similar to the previous measurements made in microglia, we calculated the longest branch and the sum of all branch lengths of each astrocyte arbor, which were depicted as maximum and total branch length, respectively. Measurements of cell arbor disclosed that maximum branch length remained without alterations between hippocampal astrocytes from control and prenatally LPS-exposed offspring (Figure 5D). Nonetheless, astrocytes from prenatally LPS-exposed offspring exhibited a ∼2-fold increase in both total branch length (Figure 5E) and the number of branches (Figure 5F). Sholl analysis demonstrated that hippocampal astrocytes from the offspring of LPS-exposed dams showed more complex arbors than in control animals (Figures 5A–C,G). Specifically, in these astrocytes, both the number of intersections between branches and Sholl rings, as well as the area under the Sholl curve were increased (Figures 5G,H). Prenatal LPS exposure triggered equivalent augmented values in the maximum number of intersections, the radius of the highest count of intersections and mean of intersections (Figures 5I–K). These findings suggest that prenatal LPS enhance the complexity of astrocyte branch arbors in the hippocampus of prenatally LPS-exposed offspring.

To unveil the contribution of Cx43 hemichannel activity in prenatal LPS-induced increase in branch arbor complexity, we injected prenatally LPS-exposed offspring mice during postnatal months with the gap19 mimetic peptide containing the cell-penetrating TAT linker (Tat-gap19; 23 mg/kg, see section Materials and methods), which crosses the blood-brain barrier (BBB) (Abudara et al., 2014). Notably, Tat-gap19 completely prevented the prenatal LPS-induced increase in diverse arbor parameters, including total branch length (Figure 5E), number of branches (Figure 5F), arbor complexity (Figure 5G), the area under the Sholl curve (Figure 5H), mean of intersections (Figure 5I), maximum number of intersections (Figure 5J), and the radius of highest count of intersections (Figure 5K). Relevantly, the inactive form of Tat-gap19 containing the I130A modification (TAT-gap19^I130A^) induced no effect on the prenatal LPS-mediated increase on astrocyte arborization (Figures 5D–K). These findings suggest that opening of Cx43 hemichannels is crucial for the prenatal LPS-mediated increment in astrocyte arbor branch complexity in the offspring hippocampus.

### The Opening of Cx43 Hemichannels Is Required for the Prenatal LPS-Mediated Reduction in Arbor Branch Complexity and Dendritic Spine Density of Hippocampal Pyramidal Neurons in the Offspring

**FIGURE 6:**
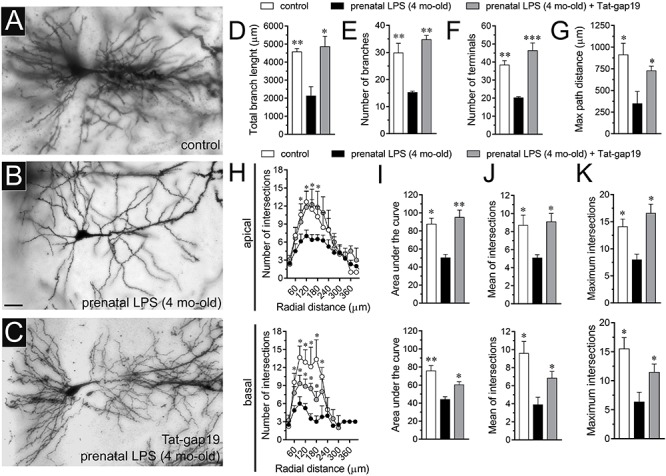
Prenatal LPS exposure increases the arborization of CA1 pyramidal neurons in the offspring by a mechanism involving the activation of Cx43 hemichannels. **(A–C)** Representative golgi (black) staining by CA1 pyramidal neurons from control offspring **(A)**, prenatally LPS-exposed offspring of 4 month old alone **(B)** or in combination with the *in vivo* administration of 23 mg/kg Tat-gap19 **(C)**. **(D–G)** Averaged data of total branch length **(D)**, number of branches **(E)**, number of terminals **(F),** and maximum path distance **(G)** by CA1 pyramidal neurons from control offspring (white bars) or prenatally LPS-exposed offspring of 4 months old alone (black bars) or in combination with the *in vivo* administration of 23 mg/kg Tat-gap19 (gray bars). ^∗^*p* < 0.05, ^∗∗^*p* < 0.01, ^∗∗∗^*p* < 0.005 versus LPS, one-way ANOVA Dunnett’s *post hoc* test, mean ± S.E.M., *n* = 3. **(H)** Averaged data of Sholl analysis by apical (upper panel) and basal (bottom panel) dendritic arbor of CA1 pyramidal neurons from control offspring (white circles) or prenatally LPS-exposed offspring of 4 months old alone (black circles) or plus the *in vivo* administration of 23 mg/kg Tat-gap19 (gray circles). ^∗^*p* < 0.05 versus LPS, two-way ANOVA Tukey’s *post hoc* test, mean ± S.E.M., *n* = 3. **(I–K)** Averaged data of area under the curve of Sholl analysis **(I)**, mean of intersections **(J)** and maximum intersection **(K)** by apical (upper panel) and basal (bottom panel) dendritic arbor of CA1 pyramidal neurons from control offspring (white bars) or prenatally LPS-exposed offspring of 4 months old alone (black bars) or plus the *in vivo* administration of 23 mg/kg Tat-gap19 (gray bars). ^∗^*p* < 0.05, ^∗∗^*p* < 0.01 versus LPS, one-way ANOVA Tukey’s *post hoc* test, mean ± S.E.M., *n* = 3. Calibration bar = 45 μm.

**FIGURE 7:**
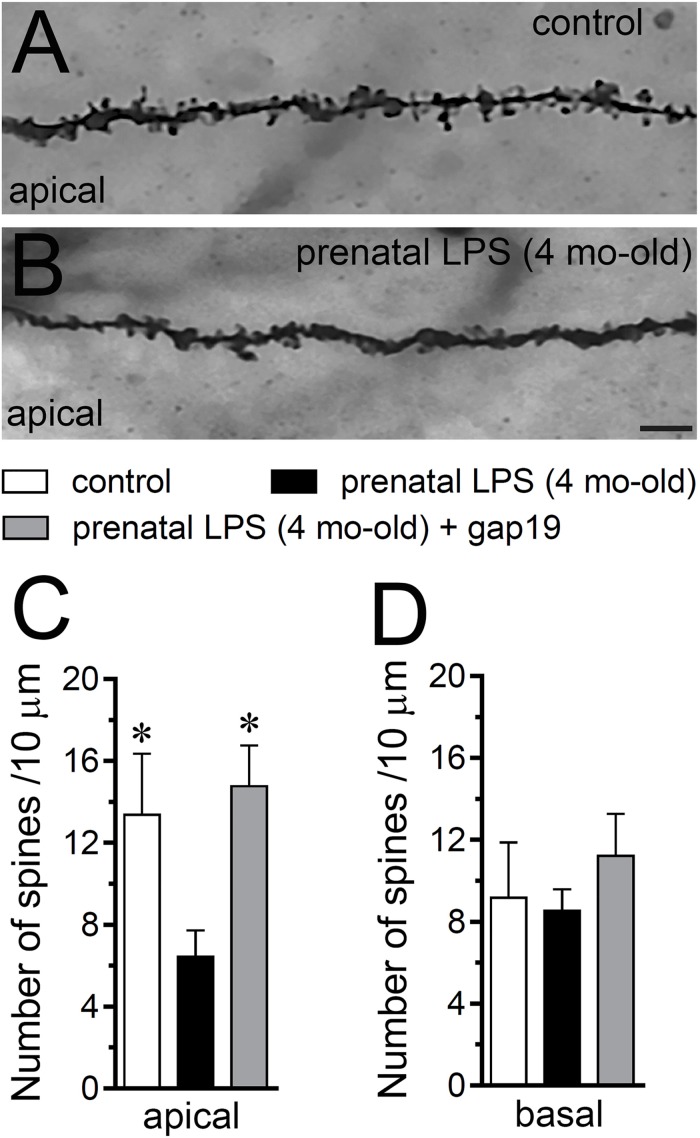
Prenatal LPS exposure increases spine density in apical but not basal dendrites of CA1 pyramidal neurons, a response based on the activation of Cx43 hemichannels. **(A,B)** Representative golgi (black) staining by apical dendrites of CA1 pyramidal neurons from control offspring **(A)** or prenatally LPS-exposed offspring of 4 months old **(B)**. **(C,D)** Averaged data of the number of apical **(A)** or basal **(B)** dendritic spines by CA1 pyramidal neurons from control offspring (white bars) or prenatally LPS-exposed offspring of 4 months old alone (black bars) or in combination with the *in vivo* administration of 23 mg/kg Tat-gap19 (gray bars). ^∗^*p* < 0.05 versus LPS, one-way ANOVA Tukey’s *post hoc* test, mean ± S.E.M., *n* = 3. Calibration bar = 3 μm.

Hippocampal synaptic dysfunction has been linked with retraction of dendrites pyramidal neurons, as well as loss of synapses in diverse neurological disorders (Luo and O’Leary, 2005; Riccomagno and Kolodkin, 2015). Nonetheless, whether prenatal LPS exposure causes dendritic retraction and spine density reduction in the hippocampus have not been studied in detail (Fernandez de Cossio et al., 2017). Here, analysis of dendritic arbor (Figures 6A–C) showed that prenatal LPS exposure caused a ∼2-fold decrease in total branch length (Figure 6D), number of branches (Figure 6E) and the number of terminals (Figure 6F) in CA1 pyramidal neurons. These neurons also exhibited a decrease in the maximum path distance between the soma and terminal dendrites when compared with their control counterparts (Figure 6G). A precise Sholl analysis underscored that CA1 pyramidal neurons from the offspring of LPS-exposed dams displayed a ∼2-fold decline in arbor complexity in both basal and apical dendrites (Figure 6H). Indeed, prenatal LPS exposure diminished the area under the Sholl curve (Figure 6I), the mean of intersections between branches and Sholl rings (Figure 6J), as well as the maximum number of intersections (Figure 6K). Of note, dendritic retraction evoked by prenatal LPS exposure was accompanied by decreased spine density in apical but not basal dendrites of CA1 pyramidal neurons (Figures 7A–D).

Consistent with what occurred in hippocampal astrocytes, *in vivo* treatment with Tat-gap19 strongly counteracted the prenatal LPS-induced decrease in arborization, although this inhibitory response was more effective in apical rather than basal dendritic arbor (Figures 6H–K). In addition, Tat-gap19 also totally blunted the prenatal LPS-induced decrease in spine density in apical dendrites of CA1 pyramidal neurons (Figure 7D). TAT-gap19^I130A^, the inactive form of Tat-gap19, did not change the prenatal LPS-mediated reduction in the neuronal pyramidal arbor or spine density (not shown). Altogether these findings argue for a crucial role of Cx43 hemichannels in the prenatal LPS-mediated reduction of dendritic arbor and spine density of CA1 pyramidal neurons.

### Cx43 Hemichannels Participate in the Prenatal LPS-Induced Neuronal Death in Hippocampal Slices

**FIGURE 8:**
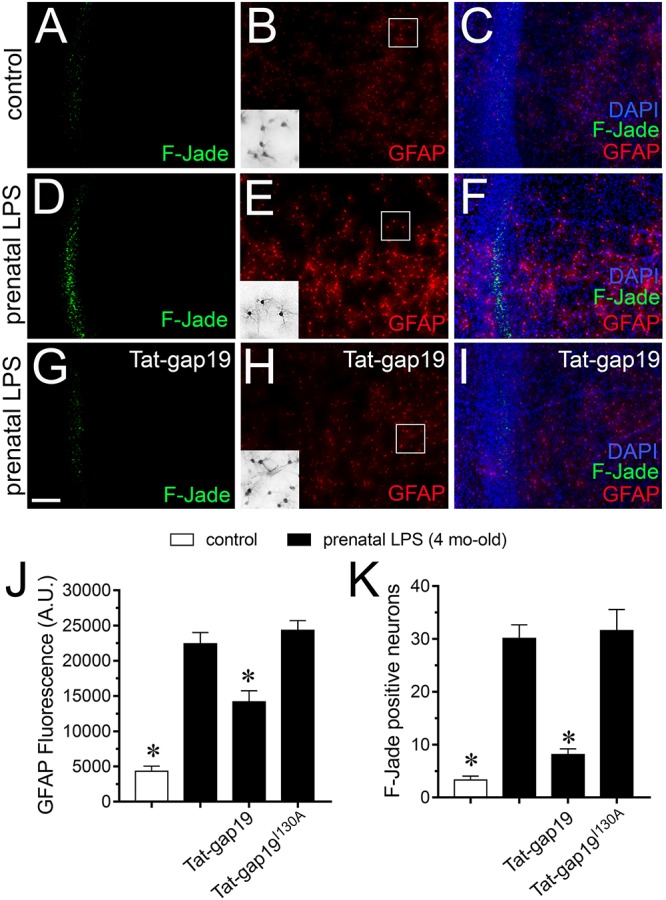
Cx43 hemichannel contributes to neuronal death evoked by prenatal LPS exposure on offspring hippocampus. **(A–I)** Representative images depicting Fluoro-Jade (F-Jade, green), GFAP (red) and DAPI (blue) staining in acute slices from control offspring **(A–C)** or prenatally LPS-exposed offspring of 4 months old alone **(D–F)** or in combination with the *in vivo* administration of 23 mg/kg Tat-gap19 **(G–I)**. Insets of gray scale GFAP staining for astrocytes are shown from the area depicted within the white squares in **(B,E,H)**. **(J)** Averaged data of GFAP fluorescence per field in acute slices from control offspring (white bars) or prenatally LPS-exposed offspring of 4 months old alone (black bars) or in combination with the *in vivo* administration of 23 mg/kg Tat-gap19 (gray bars). ^∗^*p* < 0.01 versus LPS, one-way ANOVA Tukey’s *post hoc* test, mean ± S.E.M., *n* = 3. **(K)** Averaged number of F-Jade-positive CA1 pyramidal neurons per field in acute slices from control offspring (white bars) or prenatally LPS-exposed offspring of 4 months old alone (black bars) or in combination with the *in vivo* administration of 23 mg/kg Tat-gap19 (gray bars). ^∗^*p* < 0.001 versus LPS, one-way ANOVA Tukey’s *post hoc* test, mean ± S.E.M., *n* = 3. Calibration bar = 100 μm.

It is well established that prenatal-LPS exposure triggers neuronal death in the offspring (Ling et al., 2002; Hao et al., 2010) and diverse studies have proposed that uncontrolled release of substances via opening of astrocyte Cx43 hemichannels could be toxic for neighboring neurons (Orellana et al., 2011; Yi et al., 2016). With this in mind, we evaluated if prenatal LPS exposure could induce cell death in CA1 pyramidal neurons and whether Cx43 hemichannels are involved in this process. In control offspring, most pyramidal neurons were negative for F-Jade staining (∼3 neurons/field) and most astrocytes displayed a normal grade of GFAP expression (Figures 8A–C,J,K). However, prenatally LPS-exposed offspring exhibited a ∼10-fold augment in CA1 pyramidal neurons displaying F-Jade staining, which was accompanied by a qualitative augment in GFAP reactivity (Figures 8D–F,J,K). Relevantly, *in vivo* treatment with Tat-gap19 greatly tackled both the prenatal LPS-induced F-Jade staining of CA1 pyramidal neurons (to ∼8 neurons/field) and the enchanced reactivity of GFAP (in ∼40%) in hippocampal slices (Figures 8G–K). In the presence of TAT-gap19^I130A^, the F-Jade staining by CA1 pyramidal neurons remained unaltered in prenatally LPS-exposed offspring (Figures 8J,K). The above data suggest that Cx43 hemichannels are main contributors to the hippocampal neuronal death caused by prenatal LPS exposure in the offspring.

## Discussion

In this work, we reported that prenatal LPS exposure augments the activity of astrocyte Cx43 hemichannels and Panx1 channels in the hippocampus of adult offspring mice. This enhanced channel activity occurred by a mechanism involving a microglia-dependent production of IL-1β/TNF-α and the stimulation of p38 MAPK/iNOS/[Ca^2+^]_i_-mediated pathways and purinergic/glutamatergic signaling. Noteworthy, the opening of Cx43 hemichannels affected the release of glutamate, [Ca^2+^]_i_ handling, and morphology of astrocytes, whereas also disturbed neuronal function, including the dendritic arbor and spine density, as well as survival.

Previous evidence indicates that prenatal LPS exposure triggers diverse disturbances in the offspring brain, including alterations in hippocampal-dependent synaptic plasticity and memory (Golan et al., 2005; Escobar et al., 2011; Kelley et al., 2017), as well as increased neuronal death and astrogliosis (Ling et al., 2002; Hao et al., 2010; Zager et al., 2015; Cho et al., 2018). This study suggests that part of the above-mentioned abnormalities induced by prenatal LPS exposure could take place by the persistent opening of astrocyte Cx43 hemichannels and/or Panx1 channels within the hippocampus. As assayed by Etd uptake in acute brain slices, we found that a single LPS injection during pregnancy increases the opening of Cx43 hemichannels and Panx1 channels in hippocampal astrocytes from the stratum radiatum in the offspring. These responses were prominently inhibited by Tat-L2 or gap19, whereas probenecid or ^10^panx1 showed a partial inhibitory effect. Thus, Cx43 hemichannels rather than Panx1 channels were the major responsible for the prenatal LPS-induced Etd uptake in astrocytes. The latter is consistent with the enhanced activity reported for both channels in astrocyte cultures obtained from prenatally LPS-exposed neonates (Avendano et al., 2015), as well as astrocytes from different animal pathological models such as neuropathic pain (Tonkin et al., 2018), Alzheimer’s disease (Yi et al., 2016), epileptic seizures (Santiago et al., 2011), spinal cord injury (Garre et al., 2016), and acute brain infection (Karpuk et al., 2011).

**FIGURE 9:**
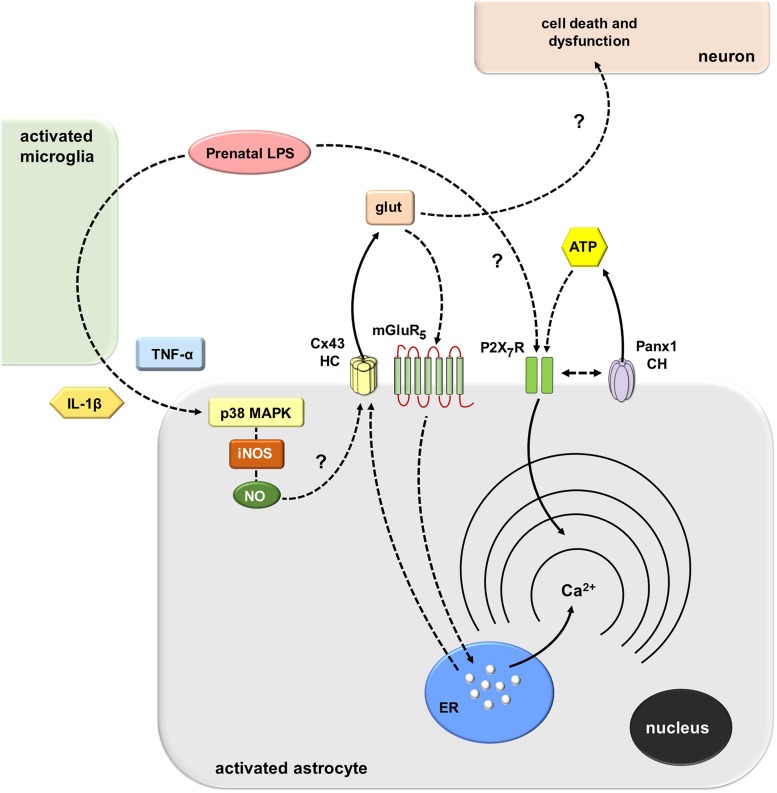
Schematic diagram showing the possible pathways involved in the prenatal LPS-induced activation of Cx43 hemichannels/Panx1 channels and its consequences for astroglial function and neuronal survival. Prenatal LPS exposure activates microglia, resulting in the release of IL-1β and TNF-α. Both cytokines stimulate astrocytes, leading to the activation of a p38MAPK/iNOS-dependent pathway and further production of NO. The latter likely induces unknown mechanisms that cause opening of Cx43 hemichannels enabling the release of glutamate. Glutamate released via Cx43 hemichannels activates mGluR_5_ receptors resulting in the stimulation of IP_3_ receptors and further release of Ca^2+^ stored in the endoplasmic reticulum. In parallel, the activation of astroglial P2X_7_ receptors lead to the opening of Panx1 channels and further release of ATP, possibly through direct protein-to-protein interactions. Relevantly, the modulation of [Ca^2+^]_i_ dynamics evoked by Cx43 hemichannels may alter astroglial morphology (not depicted), whereas the excitotoxic release of glutamate through Cx43 hemichannels may affect neuronal arborization and survival by unknown mechanisms.

How does prenatal LPS exposure trigger the opening of Cx43 hemichannels and Panx1 channels in hippocampal astrocytes *ex vivo*? Multiple lines of evidence indicate that environmental factors during early development impact the future inflammatory balance and immunity response of the offspring (Boksa, 2010). Here, we observed that prenatal LPS exposure induced a microglia-dependent long-lasting production of both IL-1β and TNF-α on offspring hippocampus. The latter response was accompanied by a profound retraction of microglia cellular processes compatible with amoeboid features typical of activated microglia. This is in agreement with the presence of activated microglia as well as with upregulated levels of IL-1β and TNF-α in the offspring’s brain of LPS-exposed dams (Boksa, 2010). In addition, our experiments showed that minocycline, a molecule that attenuates microglial activation, or inhibition of IL-1β/TNF-α signaling, dramatically suppressed the prenatal LPS-induced opening of astrocytic Cx43 hemichannels but not Panx1 channels on offspring hippocampus. These findings harmonize with prior studies showing that release of IL-1β and TNF-α from activated microglia causes the activation of astroglial Cx43 hemichannels (Retamal et al., 2007). Thereby, in our system, the opening of astroglial Cx43 hemichannels likely resulted from the IL-1β/TNF-α-mediated activation of p38 MAP kinase and further iNOS-dependent S-nitrosylation of Cx43, as has been previously described (Retamal et al., 2006; Retamal et al., 2007) (Figure 9). In accord with this hypothesis, we detected that prenatal LPS-induced astroglial Etd uptake was strongly blunted by blocking p38 MAP kinase or iNOS. In the same line, hippocampal astrocytes from prenatally LPS-exposed offspring showed increased levels of NO production, as measured by DAF-FM signal. Of note, this response was totally blunted by the inhibition of iNOS but not by blockers of Cx43 hemichannels or Panx1 channels, suggesting that these channels do not participate in astrocyte NO production caused by prenatal LPS exposure.

A cornerstone underlying the opening of Panx1 channels came from their close association with P2X_7_Rs (Dahl, 2018). Indeed, Panx1 co-immunoprecipitates with P2X_7_Rs (Pelegrin and Surprenant, 2006; Poornima et al., 2012), and proline 451 in the C-terminal tails of these receptors has been found crucial in this interaction (Iglesias et al., 2008; Sorge et al., 2012). In this context, our experiments showed that activation of P2X_7_Rs and opening of astrocytic Panx1 channels are part of the mechanism involved in the prenatal LPS-induced release of ATP on offspring hippocampus. In concordance with these findings, previous studies have described that ATP triggers its own release via P2X_7_Rs and further activation of Panx1 channels (Iglesias et al., 2009; Garre et al., 2016). The fact that Panx1 channels contribute to the prenatal LPS-induced Etd uptake and release of ATP but not glutamate is puzzling. One possibility is that despite that both channels may be permeable to Etd, they differ in their contribution to the release of ATP and glutamate in our system. Although this seems paradoxical, recent studies have demonstrated that hemichannels and pannexons do not act as freely permeable non-selective pores, but they select permeants in an isoform-specific form (Hansen et al., 2014a, b; Nielsen et al., 2017). Thus, fluorescent dye uptake cannot be employed as an indicator of permeability to ions or small biologically relevant molecules. Alternatively, other explanation to these findings may imply that another cell type, expressing functional Panx1 channels may be responsible for the Panx1-dependent release of ATP and Etd uptake found in astrocytes in the hippocampus. Regardless of its source, we can speculate that ATP may activate distant astrocytes and/or neurons in a paracrine manner, triggering Ca^2+^ responses that could rely on the reactive profile of astrocytes (Butt, 2011). If so, the stimulation of purinergic receptors could be shut down by diffusion of ATP to distant areas as well as by desensitization of P2Y receptors and degradation of extracellular ATP by exonucleases. Simultaneously, a negative feedback loop is the counteracting effect that could be evoked by ATP on Panx1 channels (Qiu and Dahl, 2009).

How does Cx43 hemichannel opening affect Ca^2+^ signaling? Hemichannels are permeable to Ca^2+^ (De Bock et al., 2012; Fiori et al., 2012). In this scenario, alterations on [Ca^2+^]_i_ handling linked to astrocyte hemichannel opening could be pivotal in the potential vicious cycle underpinning astrocyte dysfunction in the prenatally LPS-exposed offspring. Supporting this idea, we found that prenatal LPS exposure increased the basal [Ca^2+^]_i_ and the number and amplitude of spontaneous oscillations by astrocytes on offspring hippocampus. Notably, the increase of spontaneous astroglial [Ca^2+^]_i_ oscillations and their amplitude was totally blunted by BAPTA-AM but not by gap19, Tat-L2, ^10^panx1, probenecid, MTEP or SIB-1757. Moreover, the increase in basal [Ca^2+^]_i_ evoked by prenatal LPS exposure was dependent on the microglia-mediated opening of Cx43 hemichannels and subsequent stimulation of mGluR_5_, but not activation of Panx1 channels. Collectively, these data suggest that spontaneous [Ca^2+^]_i_ oscillations evoked by prenatal LPS exposure are necessary for the opening of astroglialCx43 hemichannels, which result in the release of glutamate and the subsequent downstream increase of basal [Ca^2+^]_i_ via intracellular stores (Figure 9). Consistent with this, selective blockade of PLC or IP_3_ receptors, as well as chelation of [Ca^2+^]_i_, strongly blunted the increase in basal astroglial [Ca^2+^]_i_ triggered by prenatal LPS exposure. Furthermore, prenatal LPS exposure enhanced the release of glutamate on offspring hippocampus, a response completely dependent on the opening of Cx43 hemichannels but not Panx1 channels. These data concord with previous studies showing the release of glutamate via activation of Cx43 hemichannels (Ye et al., 2003) and with the fact that mGluR_5_ controls [Ca^2+^]_i_ responses in astrocytes (Panatier and Robitaille, 2016). Previous studies have described that opening of Cx43 hemichannels is regulated by [Ca^2+^]_i_ (De Bock et al., 2012; Meunier et al., 2017). In this line, we noted that chelation of [Ca^2+^]_i_ reduced the prenatal LPS-induced Etd uptake by astrocytes on offspring hippocampus, whereas inhibition of mGluR_5_, PLC or IP_3_ receptor did not affect this response.

In the inflamed brain, among other disturbances, astrocytes and neurons undergo the remodeling of their dendritic arbor as well as several morphological changes (Luo and O’Leary, 2005; Pekny and Pekna, 2014; Riccomagno and Kolodkin, 2015). In this work, we detected for the first time that prenatal LPS exposure augments the complexity of astrocyte branch arbors on offspring hippocampus, whereas in neurons occurred the opposite. This evidence harmonizes with studies reporting that neuropathological conditions augment the arborization of hippocampal astrocytes (Beauquis et al., 2013; Chun et al., 2018) and induce dendritic retraction of CA1 pyramidal neurons (Christian et al., 2011; Burak et al., 2018). Usually, the reduction in neurite arborization lined to the loss of branching and a decline in total neurite lengths is accompanied by a decrease in synaptic number and retraction of dendritic spines (Luo and O’Leary, 2005; Rossi and Volterra, 2009). In this context, our experiments revealed that dendritic retraction of CA1 pyramidal neurons triggered by prenatal LPS occurred in parallel with a reduction in the number of apical but not basal dendritic spines in these neurons. It is not clear why the dendritic spine density of basal dendrites is not altered by prenatal LPS exposure. However, this evidence are supported by prior studies describing the layer-specific spine density of CA1 pyramidal neurons (Spruston, 2008) and the opposite regulation of this feature between apical and basal dendrites during different pathological conditions (Hyer and Glasper, 2017; Maynard et al., 2017).

Remarkably, the prenatal LPS-induced alterations on arborization and morphology of astrocytes and neurons were dramatically counteracted with the administration of a specific blocker of Cx43 hemichannels that crosses the BBB. Thus, prenatal LPS exposure elicits divergent morphological effects on offspring astrocytes and neurons that likely reflect their inflammatory status, a phenomenon in which the opening of Cx43 hemichannels seems to be crucial. Substantial levels of glutamate at the synaptic cleft could be neurotoxic under pathological conditions (Lau and Tymianski, 2010). In this context, recent evidence indicates that glutamate released by a mechanism implicating the opening of astroglial Cx43 hemichannels could reduce neuronal survival (Orellana et al., 2011; Yi et al., 2016). Here, we observed that selective inhibition of Cx43 hemichannels greatly prevents the prenatal LPS-induced death of CA1 pyramidal neurons on offspring hippocampus. The latter suggests that Cx43 hemichannels likely contribute to neuronal damage either by altering astrocyte functions (e.g., [Ca^2+^]_i_ handling) and/or through the release of excitotoxic amounts of glutamate. Supporting this idea, the prenatal LPS-induced overexpression of GFAP, [Ca^2+^]_i_ increase and release of glutamate was strongly blunted by blocking Cx43 hemichannels. Excitotoxic levels of glutamate along with a dysfunctional astroglial partnership plausibly could cause neuronal damage via osmotic and [Ca^2+^]_i_ imbalance, as well as caspase activation (Orellana et al., 2011; Moidunny et al., 2016) (Figure 9).

Our findings suggest that opening of astrocyte Cx43 hemichannels occurs at early phases of postnatal life in prenatally LPS-exposed offspring and is accompanied by hippocampal neuroinflammation, as well as diverse astrocyte and neuronal alterations in function and morphology. Future studies are needed in order to elucidate whether astroglial hemichannel/pannexon opening evoked by prenatal LPS exposure may also take place at fetal stages. We speculate that excitotoxic levels of glutamate triggered by the activation of Cx43 hemichannels may contribute to the hippocampal neurotoxicity and damage in prenatally LPS-exposed offspring. Therefore, the understanding of how astrocyte-neuron crosstalk is affected in prenatally LPS-exposed offspring is a promising avenue toward the development of common therapies for several neurological disorders observed in children born to women who had a severe infection during pregnancy.

## Data Availability Statement

The datasets generated for this study are available on request to the corresponding author.

## Ethics Statement

This study was carried out in accordance with the recommendations of “Protocolo de Cuidado y Uso Animal del Comité Ético Cienífico para el Cuidado de Animales y Ambiente.” The protocol was approved by the “Comité Ético Científico para el Cuidado de Animales y Ambiente” of the Ponificia Universidad Católica de Chile.

## Author Contributions

CC, JEO, BA, LM, CI, TA, and JAO conceived, performed, and analyzed the experiments. JAO wrote and edited the manuscript. All authors read and approved the final manuscript.

## Conflict of Interest

The authors declare that the research was conducted in the absence of any commercial or financial relationships that could be construed as a potential conflict of interest.

## Supplementary Material

FIGURE S1 Neurons do not contribute to the prenatal LPS-induced changes in astroglial function observed in the offspring hippocampus. **(A)** Averaged of DAF-FM signal fluorescence by astrocytes in acute slices from control offspring (white bar) or prenatally LPS-exposed offspring of 4 months old alone (black bars) or in combination with 0.5 μM TTX and the following pharmacological agents: 100 μM gap19, 100 μM ^10^panx1 and 1 μM L-N6. ^∗^*p* < 0.0001 versus LPS, one-way ANOVA Tukey’s *post hoc* test, mean ± S.E.M., *n* = 3. **(B)** Averaged of basal Fluo-4 signal fluorescence by hippocampal astrocytes in acute slices from control offspring (white bar) or prenatally LPS-exposed offspring of 4 months old alone (black bars) or in combination with 0.5 μM TTX and the following pharmacological agents: 50 nM minocycline, 100 μM gap19, 100 μM Tat-L2, 100 μM ^10^panx1, 500 μM Probenecid (Prob), 10 μM Bapta-AM, 5 μM U-73122, 50 μM 2-APB, 50 nM MTEP, 5 μM SIB-1757, 200 μM oATP, and 200 nM A740003. ^∗∗^*p* < 0.0001, ^∗^*p* < 0.005 versus LPS, one-way ANOVA Tukey’s *post hoc* test, mean ± S.E.M., *n* = 3. **(C)** Averaged data of glutamate release by acute hippocampal slices from control offspring (white bar) or prenatally LPS-exposed offspring of 4 months old alone (black bars) or in combination with 0.5 μM TTX and the following blockers: 100 μM gap19, 100 μM Tat-L2, 100 μM ^10^panx1, 500 μM Probenecid (Prob). ^∗^*p* < 0.0001 versus LPS, one-way ANOVA Tukey’s *post hoc* test, mean ± S.E.M., *n* = 3. **(D)** Averaged of spontaneous [Ca^2+^]_i_ oscillations by hippocampal astrocytes in acute slices from control offspring (white bar) or prenatally LPS-exposed offspring of 4 months old alone (black bars) or in combination with 0.5 μM TTX and the following pharmacological agents: 100 μM gap19, 100 μM Tat-L2, 100 μM ^10^panx1, 500 μM Probenecid (Prob), 10 μM Bapta-AM, 50 nM MTEP or 5 μM SIB-1757. ^∗∗^*p* < 0.005 versus LPS, one-way ANOVA Tukey’s *post hoc* test, mean ± S.E.M., *n* = 3. **(E)** Averaged of peak amplitude of spontaneous [Ca^2+^]_i_ oscillations by hippocampal astrocytes in acute slices from control offspring (white bar) or prenatally LPS-exposed offspring of 4 months old alone (black bars) or in combination with 0.5 μM TTX and the following pharmacological agents: 100 μM gap19, 100 μM Tat-L2, 100 μM ^10^panx1, 500 μM Probenecid (Prob), 10 μM Bapta-AM, 50 nM MTEP or 5 μM SIB-1757. ^∗∗^*p* < 0.005 versus LPS, one-way ANOVA Tukey’s *post hoc* test, mean ± S.E.M., *n* = 3.

FIGURE S2 Prenatal LPS exposure does not affect total level and distribution of Cx43 in astrocytes. **(A,B)** Representative confocal images depicting GFAP (green) and Cx43 (red) staining by astrocytes in acute slices from control offspring **(A)** or prenatally LPS-exposed offspring of 4-month old **(B)**. **(C–F)** Insets of astrocytes and examples of draws for staining analysis were taken from the area depicted within the white squares in **(A,B)**. Calibration bars: white = 120 μm; yellow = 20 μm. **(G)** Quantification of membrane, intracellular and total staining of Cx43 by astrocytes in acute slices from control offspring (white bars) or prenatally LPS-exposed offspring of 4 months old (black bars). Data were obtained from at least three independent experiments with three or more repeats each one (≥20 cells analyzed for each repeat).

## References

[B1] Abudara V. Bechberger J. Freitas-Andrade M. De Bock M. Wang N. Bultynck G. (2014). The connexin43 mimetic peptide Gap19 inhibits hemichannels without altering gap junctional communication in astrocytes. *Front. Cell. Neurosci.* 8:306. 10.3389/fncel.2014.00306 25374505 PMC4204617

[B2] Abudara V. Retamal M. A. Del Rio R. Orellana J. A. (2018). Synaptic functions of hemichannels and pannexons: a double-edged sword. *Front. Mol. Neurosci.* 11:435. 10.3389/fnmol.2018.00435 30564096 PMC6288452

[B3] Abudara V. Roux L. Dallerac G. Matias I. Dulong J. Mothet J. P. (2015). Activated microglia impairs neuroglial interaction by opening Cx43 hemichannels in hippocampal astrocytes. *Glia* 63 795–811. 10.1002/glia.22785 25643695

[B4] Agulhon C. Sun M. Y. Murphy T. Myers T. Lauderdale K. Fiacco T. A. (2012). Calcium signaling and gliotransmission in normal vs. reactive astrocytes. *Front. Pharmacol.* 3:139. 10.3389/fphar.2012.00139 22811669 PMC3395812

[B5] Ardiles A. O. Flores-Munoz C. Toro-Ayala G. Cardenas A. M. Palacios A. G. Munoz P. (2014). Pannexin 1 regulates bidirectional hippocampal synaptic plasticity in adult mice. *Front. Cell. Neurosci.* 8:326. 10.3389/fncel.2014.00326 25360084 PMC4197765

[B6] Avendano B. C. Montero T. D. Chavez C. E. Von Bernhardi R. Orellana J. A. (2015). Prenatal exposure to inflammatory conditions increases Cx43 and Panx1 unopposed channel opening and activation of astrocytes in the offspring effect on neuronal survival. *Glia* 63 2058–2072. 10.1002/glia.22877 26096155

[B7] Beauquis J. Pavia P. Pomilio C. Vinuesa A. Podlutskaya N. Galvan V. (2013). Environmental enrichment prevents astroglial pathological changes in the hippocampus of APP transgenic mice, model of Alzheimer’s disease. *Exp. Neurol.* 239 28–37. 10.1016/j.expneurol.2012.09.009 23022919

[B8] Bergdolt L. Dunaevsky A. (2018). Brain changes in a maternal immune activation model of neurodevelopmental brain disorders. *Prog. Neurobiol*. 175 1–19. 10.1016/j.pneurobio.2018.12.002 30590095 PMC6413503

[B9] Boksa P. (2010). Effects of prenatal infection on brain development and behavior: a review of findings from animal models. *Brain Behav. Immun.* 24 881–897. 10.1016/j.bbi.2010.03.005 20230889

[B10] Bradley S. J. Challiss R. A. (2012). G protein-coupled receptor signalling in astrocytes in health and disease: a focus on metabotropic glutamate receptors. *Biochem. Pharmacol.* 84 249–259. 10.1016/j.bcp.2012.04.009 22531220

[B11] Burak K. Lamoureux L. Boese A. Majer A. Saba R. Niu Y. (2018). MicroRNA-16 targets mRNA involved in neurite extension and branching in hippocampal neurons during presymptomatic prion disease. *Neurobiol. Dis.* 112 1–13. 10.1016/j.nbd.2017.12.011 29277556

[B12] Butt A. M. (2011). ATP: a ubiquitous gliotransmitter integrating neuron-glial networks. *Semin. Cell Dev. Biol.* 22 205–213. 10.1016/j.semcdb.2011.02.023 21376829

[B13] Carrigan C. N. Imperiali B. (2005). The engineering of membrane-permeable peptides. *Anal. Biochem.* 341 290–298. 10.1016/j.ab.2005.03.026 15907875

[B14] Chen B. Yang L. Chen J. Chen Y. Zhang L. Wang L. (2019). Inhibition of Connexin43 hemichannels with Gap19 protects cerebral ischemia/reperfusion injury via the JAK2/STAT3 pathway in mice. *Brain Res. Bull.* 146 124–135. 10.1016/j.brainresbull.2018.12.009 30593877

[B15] Chever O. Lee C. Y. Rouach N. (2014). Astroglial connexin43 hemichannels tune basal excitatory synaptic transmission. *J. Neurosci.* 34 11228–11232. 10.1523/JNEUROSCI.0015-14.2014 25143604 PMC6615508

[B16] Cho J. W. Jung S. Y. Kim D. Y. Chung Y. R. Choi H. H. Jeon J. W. (2018). PI3K-Akt-Wnt pathway is implicated in exercise-induced improvement of short-term memory in cerebral palsy rats. *Int. Neurourol. J.* 22 S156–S164. 10.5213/inj.1836224.112 30396265 PMC6234731

[B17] Christian K. M. Miracle A. D. Wellman C. L. Nakazawa K. (2011). Chronic stress-induced hippocampal dendritic retraction requires CA3 NMDA receptors. *Neuroscience* 174 26–36. 10.1016/j.neuroscience.2010.11.033 21108993 PMC3020251

[B18] Chun H. An H. Lim J. Woo J. Lee J. Ryu H. (2018). Astrocytic proBDNF and tonic GABA distinguish active versus reactive astrocytes in hippocampus. *Exp. Neurobiol.* 27 155–170. 10.5607/en.2018.27.3.155 30022867 PMC6050417

[B19] Contreras J. E. Sánchez H. A. Eugenin E. A. Speidel D. Theis M. Willecke K. (2002). Metabolic inhibition induces opening of unapposed connexin 43 gap junction hemichannels and reduces gap junctional communication in cortical astrocytes in culture. *Proc. Natl. Acad. Sci. U.S.A.* 99 495–500. 10.1073/pnas.012589799 11756680 PMC117588

[B20] Crespo Yanguas S. Da Silva T. C. Pereira I. V. A. Willebrords J. Maes M. Sayuri Nogueira M. (2018). TAT-Gap19 and carbenoxolone alleviate liver fibrosis in mice. *Int. J. Mol. Sci.* 19:E817. 10.3390/ijms19030817 29534516 PMC5877678

[B21] Dahl G. (2018). The Pannexin1 membrane channel: distinct conformations and functions. *FEBS Lett.* 592 3201–3209. 10.1002/1873-3468.13115 29802622

[B22] De Bock M. Wang N. Bol M. Decrock E. Ponsaerts R. Bultynck G. (2012). Connexin 43 hemichannels contribute to cytoplasmic Ca2+ oscillations by providing a bimodal Ca2+-dependent Ca2+ entry pathway. *J. Biol. Chem.* 287 12250–12266. 10.1074/jbc.M111.299610 22351781 PMC3320976

[B23] Escobar M. Crouzin N. Cavalier M. Quentin J. Roussel J. Lante F. (2011). Early, time-dependent disturbances of hippocampal synaptic transmission and plasticity after in utero immune challenge. *Biol. Psychiatry* 70 992–999. 10.1016/j.biopsych.2011.01.009 21377655

[B24] Faa G. Manchia M. Pintus R. Gerosa C. Marcialis M. A. Fanos V. (2016). Fetal programming of neuropsychiatric disorders. *Birth Defects Res. C Embryo Today* 108 207–223. 10.1002/bdrc.21139 27774781

[B25] Fernandez de Cossio L. Guzman A. Van Der Veldt S. Luheshi G. N. (2017). Prenatal infection leads to ASD-like behavior and altered synaptic pruning in the mouse offspring. *Brain Behav. Immun.* 63 88–98. 10.1016/j.bbi.2016.09.028 27697456

[B26] Fiori M. C. Figueroa V. Zoghbi M. E. Saez J. C. Reuss L. Altenberg G. A. (2012). Permeation of calcium through purified connexin 26 hemichannels. *J. Biol. Chem.* 287 40826–40834. 10.1074/jbc.M112.383281 23048025 PMC3504794

[B27] Garre J. M. Yang G. Bukauskas F. F. Bennett M. V. (2016). FGF-1 triggers pannexin-1 hemichannel opening in spinal astrocytes of rodents and promotes inflammatory responses in acute spinal cord slices. *J. Neurosci.* 36 4785–4801. 10.1523/JNEUROSCI.4195-15.2016 27122036 PMC4846674

[B28] Golan H. M. Lev V. Hallak M. Sorokin Y. Huleihel M. (2005). Specific neurodevelopmental damage in mice offspring following maternal inflammation during pregnancy. *Neuropharmacology* 48 903–917. 10.1016/j.neuropharm.2004.12.023 15829260

[B29] Gomez G. I. Falcon R. V. Maturana C. J. Labra V. C. Salgado N. Rojas C. A. (2018). Heavy alcohol exposure activates astroglial hemichannels and pannexons in the hippocampus of adolescent rats: effects on neuroinflammation and astrocyte arborization. *Front. Cell. Neurosci.* 12:472. 10.3389/fncel.2018.00472 30564103 PMC6288256

[B30] Gumusoglu S. B. Stevens H. E. (2019). Maternal inflammation and neurodevelopmental programming: a review of preclinical outcomes and implications for translational psychiatry. *Biol. Psychiatry* 85 107–121. 10.1016/j.biopsych.2018.08.008 30318336

[B31] Hansen D. B. Braunstein T. H. Nielsen M. S. Macaulay N. (2014a). Distinct permeation profiles of the connexin 30 and 43 hemichannels. *FEBS Lett.* 588 1446–1457. 10.1016/j.febslet.2014.01.036 24503060

[B32] Hansen D. B. Ye Z. C. Calloe K. Braunstein T. H. Hofgaard J. P. Ransom B. R. (2014b). Activation, permeability, and inhibition of astrocytic and neuronal large pore (hemi)channels. *J. Biol. Chem.* 289 26058–26073. 10.1074/jbc.M114.582155 25086040 PMC4176216

[B33] Hao L. Y. Hao X. Q. Li S. H. Li X. H. (2010). Prenatal exposure to lipopolysaccharide results in cognitive deficits in age-increasing offspring rats. *Neuroscience* 166 763–770. 10.1016/j.neuroscience.2010.01.006 20074621

[B34] Hyer M. M. Glasper E. R. (2017). Separation increases passive stress-coping behaviors during forced swim and alters hippocampal dendritic morphology in California mice. *PLoS One* 12:e0175713. 10.1371/journal.pone.0175713 28406977 PMC5391050

[B35] Iglesias R. Dahl G. Qiu F. Spray D. C. Scemes E. (2009). Pannexin 1: the molecular substrate of astrocyte “hemichannels”. *J. Neurosci.* 29 7092–7097. 10.1523/JNEUROSCI.6062-08.2009 19474335 PMC2733788

[B36] Iglesias R. Locovei S. Roque A. Alberto A. P. Dahl G. Spray D. C. (2008). P2X7 receptor-Pannexin1 complex: pharmacology and signaling. *Am. J. Physiol. Cell Physiol.* 295 C752–C760. 10.1152/ajpcell.00228.2008 18596211 PMC2544446

[B37] Iyyathurai J. D’hondt C. Wang N. De Bock M. Himpens B. Retamal M. A. (2013). Peptides and peptide-derived molecules targeting the intracellular domains of Cx43: gap junctions versus hemichannels. *Neuropharmacology* 75 491–505. 10.1016/j.neuropharm.2013.04.050 23664811

[B38] Johnson R. G. Le H. C. Evenson K. Loberg S. W. Myslajek T. M. Prabhu A. (2016). Connexin hemichannels: methods for dye uptake and leakage. *J. Membr. Biol.* 249 713–741. 10.1007/s00232-016-9925-y 27586664

[B39] Karpuk N. Burkovetskaya M. Fritz T. Angle A. Kielian T. (2011). Neuroinflammation leads to region-dependent alterations in astrocyte gap junction communication and hemichannel activity. *J. Neurosci.* 31 414–425. 10.1523/JNEUROSCI.5247-10.2011 21228152 PMC3089986

[B40] Kelley M. H. Wu W. W. Lei J. Mclane M. Xie H. Hart K. D. (2017). Functional changes in hippocampal synaptic signaling in offspring survivors of a mouse model of intrauterine inflammation. *J. Neuroinflammation* 14:180. 10.1186/s12974-017-0951-1 28874190 PMC5583754

[B41] Kettenmann H. Hanisch U. K. Noda M. Verkhratsky A. (2011). Physiology of microglia. *Physiol. Rev.* 91 461–553. 10.1152/physrev.00011.2010 21527731

[B42] Kim H. S. Suh Y. H. (2009). Minocycline and neurodegenerative diseases. *Behav. Brain Res.* 196 168–179. 10.1016/j.bbr.2008.09.040 18977395

[B43] Lau A. Tymianski M. (2010). Glutamate receptors, neurotoxicity and neurodegeneration. *Pflugers Arch.* 460 525–542. 10.1007/s00424-010-0809-1 20229265

[B44] Leybaert L. Lampe P. D. Dhein S. Kwak B. R. Ferdinandy P. Beyer E. C. (2017). Connexins in cardiovascular and neurovascular health and disease: pharmacological implications. *Pharmacol. Rev.* 69 396–478. 10.1124/pr.115.012062 28931622 PMC5612248

[B45] Ling Z. Gayle D. A. Ma S. Y. Lipton J. W. Tong C. W. Hong J. S. (2002). In utero bacterial endotoxin exposure causes loss of tyrosine hydroxylase neurons in the postnatal rat midbrain. *Mov. Disord.* 17 116–124. 10.1002/mds.10078 11835448

[B46] Luo L. O’Leary D. D. (2005). Axon retraction and degeneration in development and disease. *Annu. Rev. Neurosci.* 28 127–156. 10.1146/annurev.neuro.28.061604.135632 16022592

[B47] Maatouk L. Yi C. Carrillo-De Sauvage M. A. Compagnion A. C. Hunot S. Ezan P. (2019). Glucocorticoid receptor in astrocytes regulates midbrain dopamine neurodegeneration through connexin hemichannel activity. *Cell Death Differ* 26 580–596. 10.1038/s41418-018-0150-3 30006609 PMC6370798

[B48] Makinson R. Lloyd K. Rayasam A. Mckee S. Brown A. Barila G. (2017). Intrauterine inflammation induces sex-specific effects on neuroinflammation, white matter, and behavior. *Brain Behav. Immun.* 66 277–288. 10.1016/j.bbi.2017.07.016 28739513 PMC6916731

[B49] Maynard K. R. Hobbs J. W. Sukumar M. Kardian A. S. Jimenez D. V. Schloesser R. J. (2017). Bdnf mRNA splice variants differentially impact CA1 and CA3 dendrite complexity and spine morphology in the hippocampus. *Brain Struct. Funct.* 222 3295–3307. 10.1007/s00429-017-1405-3 28324222 PMC5608635

[B50] Meunier C. Wang N. Yi C. Dallerac G. Ezan P. Koulakoff A. (2017). Contribution of astroglial Cx43 hemichannels to the modulation of glutamatergic currents by D-serine in the mouse prefrontal cortex. *J. Neurosci.* 37 9064–9075. 10.1523/JNEUROSCI.2204-16.2017 28821660 PMC6596802

[B51] Moidunny S. Matos M. Wesseling E. Banerjee S. Volsky D. J. Cunha R. A. (2016). Oncostatin M promotes excitotoxicity by inhibiting glutamate uptake in astrocytes: implications in HIV-associated neurotoxicity. *J. Neuroinflammation* 13:144. 10.1186/s12974-016-0613-8 27287400 PMC4903004

[B52] Myatt D. R. Hadlington T. Ascoli G. A. Nasuto S. J. (2012). Neuromantic - from semi-manual to semi-automatic reconstruction of neuron morphology. *Front. Neuroinform.* 6:4. 10.3389/fninf.2012.00004 22438842 PMC3305991

[B53] Nielsen B. S. Alstrom J. S. Nicholson B. J. Nielsen M. S. Macaulay N. (2017). Permeant-specific gating of connexin 30 hemichannels. *J. Biol. Chem.* 292 19999–20009. 10.1074/jbc.M117.805986 28982982 PMC5723989

[B54] Orellana J. A. Froger N. Ezan P. Jiang J. X. Bennett M. V. Naus C. C. (2011). ATP and glutamate released via astroglial connexin 43 hemichannels mediate neuronal death through activation of pannexin 1 hemichannels. *J. Neurochem.* 118 826–840. 10.1111/j.1471-4159.2011.07210.x 21294731 PMC3108012

[B55] Orellana J. A. Montero T. D. Von Bernhardi R. (2013). Astrocytes inhibit nitric oxide-dependent Ca(2+) dynamics in activated microglia: involvement of ATP released via pannexin 1 channels. *Glia* 61 2023–2037. 10.1002/glia.22573 24123492

[B56] Orellana J. A. Retamal M. A. Moraga-Amaro R. Stehberg J. (2016). Role of astroglial hemichannels and pannexons in memory and neurodegenerative diseases. *Front. Integr. Neurosci.* 10:26. 10.3389/fnint.2016.00026 27489539 PMC4951483

[B57] Panatier A. Robitaille R. (2016). Astrocytic mGluR5 and the tripartite synapse. *Neuroscience* 323 29–34. 10.1016/j.neuroscience.2015.03.063 25847307

[B58] Pekny M. Pekna M. (2014). Astrocyte reactivity and reactive astrogliosis: costs and benefits. *Physiol. Rev.* 94 1077–1098. 10.1152/physrev.00041.2013 25287860

[B59] Pelegrin P. Surprenant A. (2006). Pannexin-1 mediates large pore formation and interleukin-1beta release by the ATP-gated P2X7 receptor. *EMBO J.* 25 5071–5082. 10.1038/sj.emboj.7601378 17036048 PMC1630421

[B60] Perea G. Navarrete M. Araque A. (2009). Tripartite synapses: astrocytes process and control synaptic information. *Trends Neurosci.* 32 421–431. 10.1016/j.tins.2009.05.001 19615761

[B61] Poornima V. Madhupriya M. Kootar S. Sujatha G. Kumar A. Bera A. K. (2012). P2X7 receptor-pannexin 1 hemichannel association: effect of extracellular calcium on membrane permeabilization. *J. Mol. Neurosci.* 46 585–594. 10.1007/s12031-011-9646-8 21932038

[B62] Qiu F. Dahl G. (2009). A permeant regulating its permeation pore: inhibition of pannexin 1 channels by ATP. *Am. J. Physiol. Cell Physiol.* 296 C250–C255. 10.1152/ajpcell.00433.2008 18945939 PMC2643853

[B63] Retamal M. A. Cortes C. J. Reuss L. Bennett M. V. Saez J. C. (2006). S-nitrosylation and permeation through connexin 43 hemichannels in astrocytes: induction by oxidant stress and reversal by reducing agents. *Proc. Natl. Acad. Sci. U.S.A.* 103 4475–4480. 10.1073/pnas.0511118103 16537412 PMC1450196

[B64] Retamal M. A. Froger N. Palacios-Prado N. Ezan P. Saez P. J. Saez J. C. (2007). Cx43 hemichannels and gap junction channels in astrocytes are regulated oppositely by proinflammatory cytokines released from activated microglia. *J. Neurosci.* 27 13781–13792. 10.1523/jneurosci.2042-07.2007 18077690 PMC6673621

[B65] Riccomagno M. M. Kolodkin A. L. (2015). Sculpting neural circuits by axon and dendrite pruning. *Annu. Rev. Cell Dev. Biol.* 31 779–805. 10.1146/annurev-cellbio-100913-013038 26436703 PMC4668927

[B66] Rossi D. Volterra A. (2009). Astrocytic dysfunction: insights on the role in neurodegeneration. *Brain Res. Bull.* 80 224–232. 10.1016/j.brainresbull.2009.07.012 19631259

[B67] Rousset C. I. Chalon S. Cantagrel S. Bodard S. Andres C. Gressens P. (2006). Maternal exposure to LPS induces hypomyelination in the internal capsule and programmed cell death in the deep gray matter in newborn rats. *Pediatr. Res.* 59 428–433. 10.1203/01.pdr.0000199905.08848.55 16492984

[B68] Salameh A. Blanke K. Dhein S. (2013). Mind the gap! Connexins and pannexins in physiology, pharmacology and disease. *Front. Pharmacol.* 4:144 10.3389/fphar.2013.00144 PMC3836048 24312055

[B69] Santello M. Toni N. Volterra A. (2019). Astrocyte function from information processing to cognition and cognitive impairment. *Nat. Neurosci.* 22 154–166. 10.1038/s41593-018-0325-8 30664773

[B70] Santiago M. F. Veliskova J. Patel N. K. Lutz S. E. Caille D. Charollais A. (2011). Targeting pannexin1 improves seizure outcome. *PLoS One* 6:e25178. 10.1371/journal.pone.0025178 21949881 PMC3175002

[B71] Schaafsma W. Basterra L. B. Jacobs S. Brouwer N. Meerlo P. Schaafsma A. (2017). Maternal inflammation induces immune activation of fetal microglia and leads to disrupted microglia immune responses, behavior, and learning performance in adulthood. *Neurobiol. Dis.* 106 291–300. 10.1016/j.nbd.2017.07.017 28751257

[B72] Schindelin J. Arganda-Carreras I. Frise E. Kaynig V. Longair M. Pietzsch T. (2012). Fiji: an open-source platform for biological-image analysis. *Nat. Methods* 9 676–682. 10.1038/nmeth.2019 22743772 PMC3855844

[B73] Sholl D. A. (1953). Dendritic organization in the neurons of the visual and motor cortices of the cat. *J. Anat.* 87 387–406. 13117757 PMC1244622

[B74] Sorge R. E. Trang T. Dorfman R. Smith S. B. Beggs S. Ritchie J. (2012). Genetically determined P2X7 receptor pore formation regulates variability in chronic pain sensitivity. *Nat. Med.* 18 595–599. 10.1038/nm.2710 22447075 PMC3350463

[B75] Spruston N. (2008). Pyramidal neurons: dendritic structure and synaptic integration. *Nat. Rev. Neurosci.* 9 206–221. 10.1038/nrn2286 18270515

[B76] Stehberg J. Moraga-Amaro R. Salazar C. Becerra A. Echeverria C. Orellana J. A. (2012). Release of gliotransmitters through astroglial connexin 43 hemichannels is necessary for fear memory consolidation in the basolateral amygdala. *FASEB J.* 26 3649–3657. 10.1096/fj.11-198416 22665389

[B77] Tonkin R. S. Bowles C. Perera C. J. Keating B. A. Makker P. G. S. Duffy S. S. (2018). Attenuation of mechanical pain hypersensitivity by treatment with Peptide5, a connexin-43 mimetic peptide, involves inhibition of NLRP3 inflammasome in nerve-injured mice. *Exp. Neurol.* 300 1–12. 10.1016/j.expneurol.2017.10.016 29055716

[B78] Walrave L. Vinken M. Albertini G. De Bundel D. Leybaert L. Smolders I. J. (2016). Inhibition of Connexin43 hemichannels impairs spatial short-term memory without affecting spatial working memory. *Front. Cell. Neurosci.* 10:288. 10.3389/fncel.2016.00288 28066184 PMC5168429

[B79] Wang N. De Vuyst E. Ponsaerts R. Boengler K. Palacios-Prado N. Wauman J. (2013). Selective inhibition of Cx43 hemichannels by Gap19 and its impact on myocardial ischemia/reperfusion injury. *Basic Res. Cardiol.* 108:309. 10.1007/s00395-012-0309-x 23184389 PMC3666173

[B80] Ye Z. C. Wyeth M. S. Baltan-Tekkok S. Ransom B. R. (2003). Functional hemichannels in astrocytes: a novel mechanism of glutamate release. *J. Neurosci.* 23 3588–3596. 10.1523/jneurosci.23-09-03588.2003 12736329 PMC6742182

[B81] Yi C. Mei X. Ezan P. Mato S. Matias I. Giaume C. (2016). Astroglial connexin43 contributes to neuronal suffering in a mouse model of Alzheimer’s disease. *Cell Death Differ.* 23 1691–1701. 10.1038/cdd.2016.63 27391799 PMC5041199

[B82] Zager A. Peron J. P. Mennecier G. Rodrigues S. C. Aloia T. P. Palermo-Neto J. (2015). Maternal immune activation in late gestation increases neuroinflammation and aggravates experimental autoimmune encephalomyelitis in the offspring. *Brain Behav. Immun.* 43 159–171. 10.1016/j.bbi.2014.07.021 25108214

