# Supplementary figures and images for: The Opening of Connexin 43 Hemichannels Alters Hippocampal Astrocyte Function and Neuronal Survival in Prenatally LPS-Exposed Adult Offspring
Source: Front Cell Neurosci. 2019 Oct 11;13:460. doi: 10.3389/fncel.2019.00460 (PMC6797550; doi:10.3389/fncel.2019.00460)

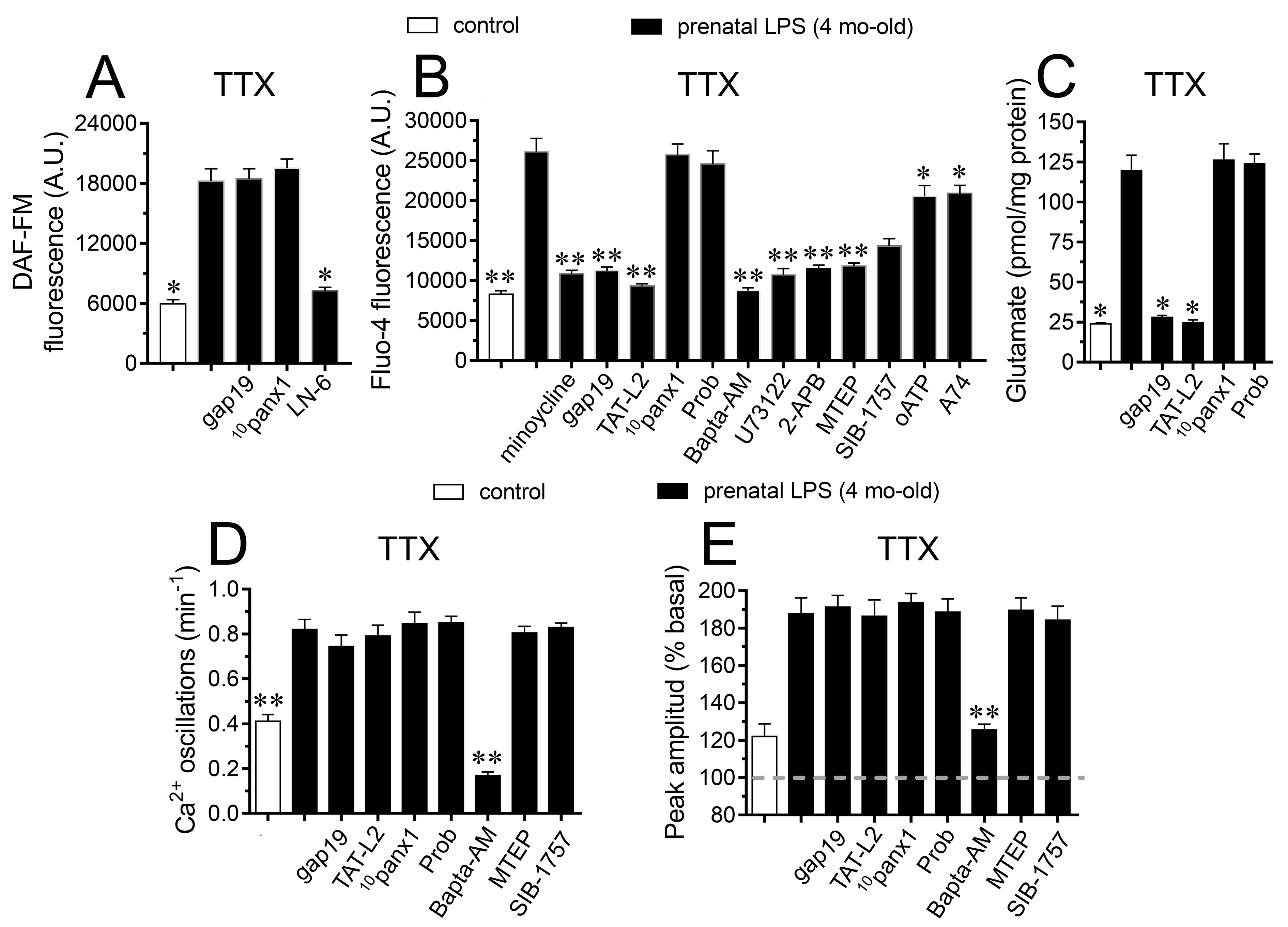

Supplement: FIGURE S1 — Neurons do not contribute to the prenatal LPS-induced changes in astroglial function observed in the offspring hippocampus. (A) Averaged of DAF-FM signal fluorescence by astrocytes in acute slices from control offspring (white bar) or prenatally LPS-exposed offspring of 4 months old alone (black bars) or in combination with 0.5 μM TTX and the following pharmacological agents: 100 μM gap19, 100 μM 10panx1 and 1 μM L-N6. ∗p < 0.0001 versus LPS, one-way ANOVA Tukey’s post hoc test, mean ± S.E.M., n = 3. (B) Averaged of basal Fluo-4 signal fluorescence by hippocampal astrocytes in acute slices from control offspring (white bar) or prenatally LPS-exposed offspring of 4 months old alone (black bars) or in combination with 0.5 μM TTX and the following pharmacological agents: 50 nM minocycline, 100 μM gap19, 100 μM Tat-L2, 100 μM 10panx1, 500 μM Probenecid (Prob), 10 μM Bapta-AM, 5 μM U-73122, 50 μM 2-APB, 50 nM MTEP, 5 μM SIB-1757, 200 μM oATP, and 200 nM A740003. ∗∗p < 0.0001, ∗p < 0.005 versus LPS, one-way ANOVA Tukey’s post hoc test, mean ± S.E.M., n = 3. (C) Averaged data of glutamate release by acute hippocampal slices from control offspring (white bar) or prenatally LPS-exposed offspring of 4 months old alone (black bars) or in combination with 0.5 μM TTX and the following blockers: 100 μM gap19, 100 μM Tat-L2, 100 μM 10panx1, 500 μM Probenecid (Prob). ∗p < 0.0001 versus LPS, one-way ANOVA Tukey’s post hoc test, mean ± S.E.M., n = 3. (D) Averaged of spontaneous [Ca2+]i oscillations by hippocampal astrocytes in acute slices from control offspring (white bar) or prenatally LPS-exposed offspring of 4 months old alone (black bars) or in combination with 0.5 μM TTX and the following pharmacological agents: 100 μM gap19, 100 μM Tat-L2, 100 μM 10panx1, 500 μM Probenecid (Prob), 10 μM Bapta-AM, 50 nM MTEP or 5 μM SIB-1757. ∗∗p < 0.005 versus LPS, one-way ANOVA Tukey’s post hoc test, mean ± S.E.M., n = 3. (E) Averaged of peak amplitude of spontaneous [Ca2+]i oscillations by h [file Image_1.JPEG]

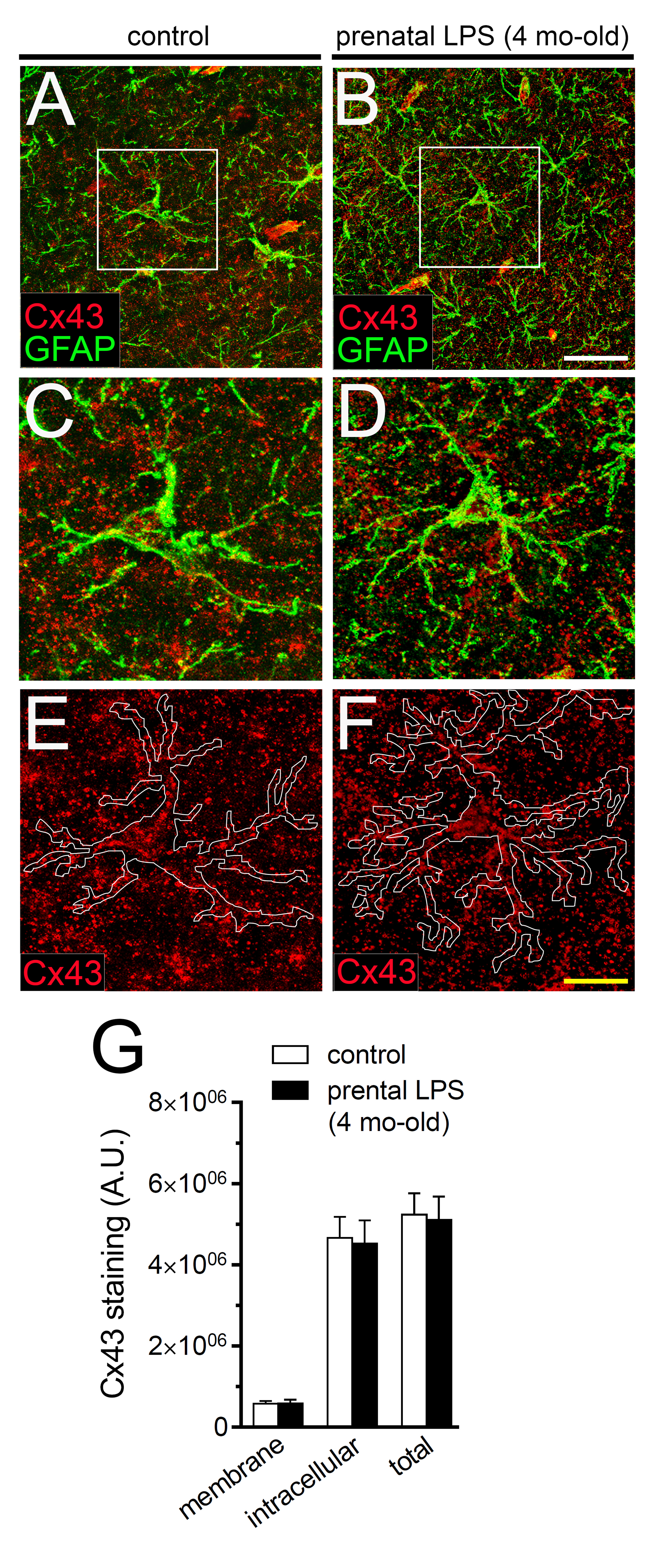

Supplement: FIGURE S2 — Prenatal LPS exposure does not affect total level and distribution of Cx43 in astrocytes. (A,B) Representative confocal images depicting GFAP (green) and Cx43 (red) staining by astrocytes in acute slices from control offspring (A) or prenatally LPS-exposed offspring of 4-month old (B). (C–F) Insets of astrocytes and examples of draws for staining analysis were taken from the area depicted within the white squares in (A,B). Calibration bars: white = 120 μm; yellow = 20 μm. (G) Quantification of membrane, intracellular and total staining of Cx43 by astrocytes in acute slices from control offspring (white bars) or prenatally LPS-exposed offspring of 4 months old (black bars). Data were obtained from at least three independent experiments with three or more repeats each one (≥20 cells analyzed for each repeat). [file Image_2.JPEG]
